# Host species-specific gene expression by a widespread and flexible chemosynthetic symbiont

**DOI:** 10.1093/ismejo/wrag065

**Published:** 2026-03-24

**Authors:** A Carlotta Kück, Lukas Leibrecht, Isidora Morel-Letelier, Olivier Gros, Laetitia G E Wilkins, Benedict Yuen-Simović, Jillian M Petersen

**Affiliations:** Eco-Evolutionary Interactions Group, Max Planck Institute for Marine Microbiology, Celsiusstraße 1, 28359 Bremen, Germany; Biogeochemistry Department, Max Planck Institute for Marine Microbiology, Celsiusstraße 1, 28359 Bremen, Germany; Centre for Microbiology and Environmental Systems Science, Department for Microbiology and Ecosystem Science, Division of Microbial Ecology, University of Vienna, Djerassiplatz 1, 1030 Vienna, Austria; Vienna Doctoral School of Microbiology and Environmental Science, University of Vienna, Djerassiplatz 1, 1030 Vienna, Austria; Eco-Evolutionary Interactions Group, Max Planck Institute for Marine Microbiology, Celsiusstraße 1, 28359 Bremen, Germany; ECR Research Group for Ecological Genomics, Max Planck Institute for Marine Microbiology, Celsiusstraße 1, 28359 Bremen, Germany; Institut de Systématique, Evolution, Biodiversité, Museum National d’Histoire Naturelle, CNRS, Sorbonne Université, EPHE, Université des Antilles, 97110 Pointe-à-Pitre, France; Eco-Evolutionary Interactions Group, Max Planck Institute for Marine Microbiology, Celsiusstraße 1, 28359 Bremen, Germany; Eco-Evolutionary Interactions Group, Max Planck Institute for Marine Microbiology, Celsiusstraße 1, 28359 Bremen, Germany; Centre for Microbiology and Environmental Systems Science, Department for Microbiology and Ecosystem Science, Division of Microbial Ecology, University of Vienna, Djerassiplatz 1, 1030 Vienna, Austria; Vienna Doctoral School of Microbiology and Environmental Science, University of Vienna, Djerassiplatz 1, 1030 Vienna, Austria

**Keywords:** animal-microbe interactions, symbiosis, marine, lucinid, metatranscriptomics, isotopes, flexibility, biodiversity, adaptation

## Abstract

Associations with microbial symbionts shape the ecology and evolution of almost all eukaryotes. One of their defining features is their specificity, but despite this, many symbioses show a degree of flexibility, with some symbiont species capable of colonizing multiple (often closely related) host species. Although widespread, the functional and evolutionary consequences of flexibility in host-symbiont pairings are poorly understood. Bivalves from the diverse, globally distributed, and ecologically important family Lucinidae are ideal for investigating this, as multiple host species can associate with the same symbiont species, often at the same location. We used metatranscriptomics to investigate the molecular responses of one symbiont species, *Candidatus* Thiodiazotropha endolucinida, in association with three different host species that co-occur in seagrass meadows in the Caribbean Sea. In replicated experiments, we identified host species-specific patterns of symbiont gene expression including those for key functions such as carbon fixation, cell division, and sulfide oxidation. Our work shows that the symbiont consistently responds in different ways to association with different host species. Because all samples were collected at the same site on the same day, and were thus exposed to the same environmental conditions, these differences are likely driven by host rather than environmental factors. In addition, host species had significantly different carbon isotope signatures, which were consistent with distinct modes of host-microbe interaction indicated by transcriptomics. Our results show that not only symbiont genotype, but also symbiont phenotype may enable coexistence of closely related host species, demonstrating the power of symbiosis in promoting and maintaining biodiversity.

## Introduction

Symbiosis has shaped the ecology and evolution of virtually all eukaryotic organisms [1, 2]. These partnerships facilitate resource exchange, enable adaptation to extreme or resource-limited environments, protect against predators and pathogens, and expand ecological niches [3–6]. Symbiotic associations are typically highly selective with distinct host species associating with specific symbiont genotypes [7–9]. This specificity in host-symbiont pairings complicates efforts to disentangle the individual contributions of host and symbiont to holobiont phenotype and function [10]. Despite this specificity, there are examples of flexibility on either the host or the symbiont side, meaning that sometimes, one host species can establish associations with multiple different symbiont species, or the same symbiont species can associate with multiple different host species [11–13]. These cases provide unique opportunities to uncover the molecular principles that underpin symbiont responses and adaptation to living in a host [11, 14, 15].

Chemosynthetic symbioses in marine ecosystems are a prime example of essential host-microbe partnerships. The autotrophic microbial symbionts enable their animal hosts to successfully colonize environments where conventional food sources are scarce, such as the dark deep sea [16, 17] and even toxic habitats, as symbionts can decrease the toxicity of substances such as hydrogen sulfide [18, 19]. The Lucinidae family is the most species-rich group of bivalves known to host chemosynthetic symbionts [20, 21]. These clams (lucinids) are an emerging experimental system for investigating fundamental principles of symbiotic interactions. The clams inhabit sulfide-rich sediments and maintain access to oxygen, carbon dioxide, and nutrients from the water column through sediment “tunnels” [22, 23]. Their gammaproteobacterial symbionts allow them to thrive across a wide range of habitats globally, including seagrass meadows, coral reef sediments, mangrove ecosystems, and deep-sea hydrothermal vents [24]. Lucinids form obligate associations with sulfur-oxidizing endosymbionts, primarily *Candidatus* Thiodiazotropha spp*.*, that reside in specialized gill epithelial cells called bacteriocytes [24–28]. Symbionts oxidize reduced sulfur compounds from the environment to fix carbon, providing nutrition to their host [29, 30]. Although lucinids are mixotrophic organisms capable of filter feeding, their nutrition relies mostly on their horizontally acquired autotrophic sulfur-oxidizing endosymbiotic bacteria [31–35].

All *Ca.* Thiodiazotropha symbionts, for which genome sequences are available, share core functions such as sulfur oxidation via the Sox, Dsr, and Sqr systems, and carbon fixation via the Calvin–Bassham–Benson cycle [24, 25, 36–43]. Other metabolic pathways vary. One of the first symbiont species sequenced, *Ca.* Thiodiazotropha endolucinida, is characterized by its diverse genomic potential for core functions of sulfur, nitrogen, and carbon metabolism [24, 25, 40]. Unlike many other species from this genus, its genome encodes both Form I and II RuBisCO enzymes, potentially enabling efficient carbon fixation across a broader range of environmental conditions, as both forms have different CO_2_ affinities [24, 44]. This species possesses *nif* genes for nitrogen fixation, the *narGHJI* operon for nitrate respiration under low-oxygen conditions, and enzymes for denitrification, enabling use of atmospheric nitrogen, and energy production in oxygen-depleted environments, respectively [25]. Furthermore, it links sulfur oxidation with nitrate respiration, providing a competitive advantage in habitats where oxygen and sulfur concentrations are variable [24]. This metabolic flexibility potentially allows this symbiont species to thrive in a much wider range of conditions than relatives that do not encode such an extensive metabolic repertoire.

Consistent with its genomic flexibility, *Ca.* T. endolucinida has been found in association with several lucinid host species, raising the possibility that its broad genomic toolkit may underpin its ability to colonize multiple host species through upregulation of distinct pathways when in association with distinct hosts. Although the genomic versatility of lucinid symbionts is well documented [24, 25, 40], gene expression in different host contexts remains unexplored. Our aim was therefore to test this theory by comparing gene expression of *Ca.* T. endolucinida in association with different lucinid species.

In the Caribbean Sea, *Ca.* T. endolucinida associates with three distinct lucinid species: *Codakia orbicularis* (Linnaeus, 1758) and *Ctena imbricatula* (C. B. Adams, 1845) from the subfamily Codakiinae, and *Clathrolucina costata* (A. d’Orbigny, 1846) from the subfamily Lucininae [34, 45]. All three host species inhabit the same seagrass bed in Guadeloupe, dominated by two seagrass species *Thalassia testudinum* K.D.Koenig, 1805 and *Syringodium filiforme* Kützing, 1860, allowing us the unique opportunity to understand symbiont transcriptomic responses to association with different host species and controlling for environmental conditions. We examined metagenomes and metatranscriptomes of symbionts in these three different host species sampled on the same day and in different seasons. To complement our transcriptomic analysis, we incorporated natural abundance stable isotope data.

## Materials and methods

### Study site, sample collection, and sample processing

**Figure 1:**
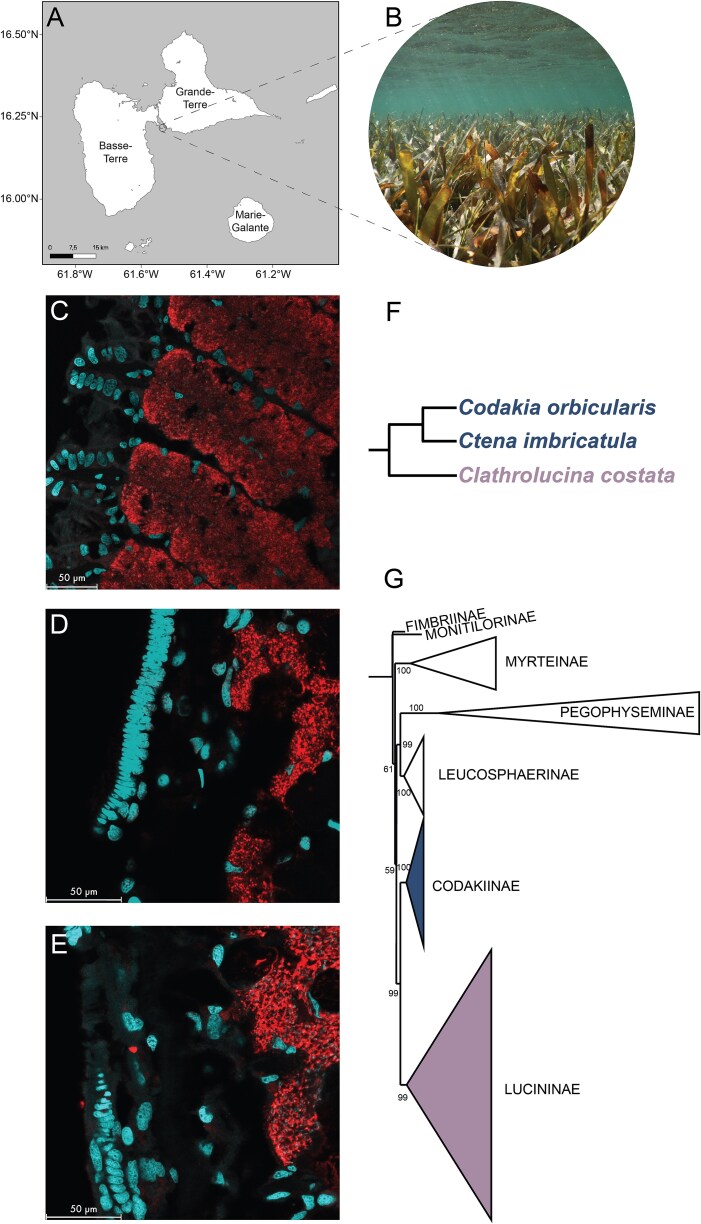
Three different lucinid species were sampled on the same day in a seagrass bed on Guadeloupe, harboring the same symbiont species: *Ca.* Thiodiazotropha endolucinida. (A, B) Sampling took place in a seagrass bed of *T. testudinum* and *S. filiforme* in Guadeloupe (French West Indies, Caribbean). Spatial distribution of *Ca.* T. endolucinida in gills of *Co. orbicularis* (C), *Ct. imbricatula* (D), and *Cl. costata* (E) visualized with FISH. Red: hybridization signal of *Ca.* T. endolucinida–specific Cy3-labeled probe, turquoise: DAPI-labeled nuclei. (F) Schematic representation of the phylogenetic relationship of *Co. orbicularis, Ct. imbricatula,* and *Cl. costata* based on the most recent taxonomic study [21] (G) Cartoon representation of Bayesian tree showing subfamily relationships of Lucinidae adapted based on the latest host phylogeny [21].

Lucinid clams were collected by hand in a mixed seagrass bed dominated by *T. testudinum* and *S. filiforme* (16.214483°N, −61.535543°W, Guadeloupe, Lesser Antilles; Fig. 1A) at sediment depths of 5-10 cm. On 2 May 2023, a total of 13 specimens were sampled, comprising 5 *Ct. imbricatula*, 5 *Cl. costata,* and 3 *Co. orbicularis* individuals. These three lucinid species differ in sizes with *Co. orbicularis* being the largest species with shell lengths of 100 mm, *Ct. imbricatula* mid-sized with shell lengths of 29 mm, and *Cl. costata* the smallest with 15.2 mm shell lengths [45]. The individuals sampled for this study fell within this size range. Bivalves were dissected on-site, with one gill from each specimen preserved in RNAlater (Thermo Fisher Scientific, Waltham, MA, USA) for metagenomic and metatranscriptomic analyses. Samples were initially stored at −80°C, transported to Bremen in an insulated container with dry ice and then stored until extractions at −80°C. The other gill was dried overnight at 45°C for isotopic analyses.

In addition to the specimens collected in May 2023, we incorporated RNA-Seq data from other lucinid specimens sampled at the same location during different time periods, specifically March 2021, May 2022, and March 2023. In March 2021, *Ct. imbricatula* (*n* = 8) and *Co. orbicularis* (*n* = 5), in May 2022, *Cl. costata* (*n* = 3), and in March 2023, *Ct. imbricatula* (*n* = 2) and *Co. orbicularis* (*n* = 1) were sampled. These additional samples were collected and processed using identical protocols but were part of different sequencing projects. They were included to investigate whether observed gene expression patterns are consistent. Detailed information on sampling and sample processing can be found in the supplements (Table S1). The map of the sampling location (Fig. 1D) was plotted using QGIS (v.3.34.3) [46] with a dataset from geoBoundaries [47] for geographic boundaries.

### Nucleic acid extraction and sequencing

#### Metatranscriptome

RNA extraction and rRNA depletion were carried out as described elsewhere [48]. Briefly, a whole, a half, and a quarter gill ctenidium were excised from *Cl. costata*, *Ct. imbricatula*, and *Co. orbicularis,* respectively, for RNA extraction. These differences in sample sizes were due to the vastly different sizes of the lucinid species in relation to the extraction protocols tissue input (10–100 mg wet tissue weight). The tissues were homogenized in TRIzol reagent (catalog number 15596018, Thermo Fisher Scientific) using bead-beating in a FastPrep (MP Biomedical), followed by total RNA extraction, DNase treatment, and purification as described previously [48]. Ribosomal RNA depletion was carried out using the Pan-Bacteria riboPOOL kit and a custom host-specific riboPOOL kit (siTOOLs Biotech). RNA concentration was measured with Qubit RNA broad-range and high-sensitivity Assay Kits (Thermo Fisher Scientific, USA), and quality was assessed with an Agilent 2100 Bioanalyzer with the Eukaryote Total RNA Nano kit (Agilent Technologies, USA). Following rRNA depletion, the eluted RNA, predominantly mRNA, was concentrated using 2.5 volumes of AMPure RNAClean XP Beads (Beckman Coulter, USA) and the entire concentrated RNA was used for metatranscriptomic library preparation. Libraries were prepared using the NEBNext Ultra II RNA Library Prep Kit for Illumina (catalog number E7770, New England Biolabs) and sequenced on a NextSeq 2000 System (Illumina) at the Max Planck-Genome-Centre Cologne, Germany to produce 2 × 150 bp paired-end reads.

#### Metagenome sequencing

##### DNA extraction

DNA was extracted using the DNeasy Blood & Tissue Kit (Qiagen, cat no: 69504), following the manufacturer’s protocol with two modifications. Proteinase K digestion at 56°C was extended to 24 h with shaking at 400 rpm. Proteinase K digestion was assessed visually, and if incomplete, an additional 20 μl of Proteinase K was added, and samples were incubated for a further 6–12 h. A second elution step was performed using the same eluate reapplied to the membrane, followed by a further 10-min incubation and centrifugation (6000 × *g*, 1 min). Eluted DNA was stored at 4°C. DNA quantity was assessed with the Qubit 1× double-stranded deoxyribonucleic acid (dsDNA) BR Assay Kit (Thermo Fisher Scientific), and quality control was performed using an Agilent 4200 TapeStation with Genomic DNA ScreenTape (Agilent Technologies, USA). Metagenome sequencing libraries were constructed using the Nextera LITE DNA library preparation kit [48], and 1 ng of genomic DNA was processed for sequencing. Sequencing of 2 × 150 bp paired-end reads was carried out on the NextSeq 2000 System at the Max Planck Genome-Centre Cologne.

##### Estimation of symbiont relative abundance and host–symbiont ratio

In seagrass beds from Guadeloupe, two potential symbiont species, *Ca.* T. endolucinida and *Ca.* T. fergusoni, have been shown to associate with lucinid bivalves. So far, *Ca.* T. fergusoni was only shown to associate with *Co. orbicularis* and *Ct. imbricatula*, but *Ca.* T. endolucinida is known to associate with all three lucinid species investigated here [24, 25]. To quantify the relative abundance of the two potential symbionts in metagenomic libraries, we performed competitive mapping using BBMap (v38.90) [49]. Metagenomic reads were trimmed and filtered to a minimum length of 100 bp, and adapters and PhiX contamination were removed using BBDuk (BBMap v37.82) [49]. Clean reads from each sample were mapped competitively against reference genomes of both symbionts (*-minid00.95 -ambiguous = random*). Read counts per reference were obtained with SAMtools (v.1.3.1) [50]. To normalize for sequencing depth, we calculated the total number of read pairs per library directly from the interleaved FASTQ files. Relative abundance for each symbiont was then computed as:


\begin{align*} Relative\ abundance= &symbiont\ species\ specific\ read s/ total\\ &symbiont\ read\ pairs \end{align*}

To independently estimate host-symbiont ratios from metagenomic libraries, small subunit (SSU) rRNA reads were extracted and analyzed using phyloFlash [50]. Small subunit read extraction, assembly with SPAdes, and taxonomic classification of both assembled and unassembled reads were performed using the phyloFlash pipeline. Taxonomic assignments were evaluated, and due to limitations in database resolution (SILVA 138.1), only SSU rRNA sequences reconstructed through assembly were retained for host-symbiont quantification. Relative host and symbiont abundance per library was quantified using the phyloFlash-reported read coverage values for each SSU rRNA contig. Host-symbiont ratios were calculated for each library by dividing the summed host SSU contig coverage by the summed symbiont SSU contig coverage. Normality of host-symbiont ratio distributions within each host species was assessed using a Shapiro-Wilk test, and homogeneity of variance across host species was evaluated using Levene’s test. As assumptions were met, differences in host-symbiont ratios among host species were tested using one-way Analysis of Variance (ANOVA) followed by Tukey’s Honest Significant Differences (Tukey HSD) post hoc pairwise comparisons. All visualizations were generated in R (version 4.4.0) using ggplot2 [51].

##### MAG recovery and taxonomic classification

Clean reads were assembled into contigs using SPAdes v4.1.0 [52] (*--meta*, *-k 21,31,41,51,61,71,81,91*). Differential coverage data required for binning were generated using BWA-MEM v0.7.18 [53], SAMtools v1.21 [54], and the *pileup.sh* script from BBMap v37.82 [49]. Contigs with a minimum length of 1000 bp were binned with MetaBAT2 v2.17 [55], Binsanity v0.5.4 [56], and MaxBin2 v2.2.7 [57]. Resulting bins were aggregated and dereplicated using DAS Tool v1.1.7 [58]. Bin quality was assessed using CheckM2 v1.0.1 [59], and bins were considered to be high-quality metagenome-assembled genomes (MAGs) when completeness was >90% and contamination <5%. Taxonomic classification of high-quality MAGs was determined by calculating the Average Nucleotide Identity (ANI), with fastANI v1.33 [60], against previously published symbiont MAGs of *Ca.* T. endolucinida (SAMN40439154, GCA_040792425.1) and *Ca.* T. fergusoni (SAMN40439166, GCA_040791165.1). Newly recovered high-quality MAGs and publicly available MAGs of *Ca.* T. endolucinida and *Ca.* T. fergusoni from lucinids sampled in Guadeloupe were used to infer a symbiont phylogenomic tree with the genome of *Ca.* Sedimenticola endophacoides as an outgroup. The outgroup species is also a lucinid symbiont associated with the host species *Phacoides pectinatus* (Gmelin, 1791), which is found in mangrove sediments on Guadeloupe [24]. The GTDB-Tk v2.3.2 classify workflow (r214) [61–64] was used to obtain a concatenated alignment of 120 conserved bacterial marker genes, and the maximum likelihood tree was inferred from the alignment using IQ-Tree v2.3.6 [65–67] (*-m MFP*, *-B 10000, --alrt 10 000*). The tree was visualized using the Interactive Tree Of Life (iTOL) [68].

#### Reference genome of *Ca.* T. endolucinida

##### Sample collection

The “P32” *Ca.* T. endolucinida reference genome was obtained from a *Ct. imbricatula* individual collected in Guadeloupe in August 2021 and kept in an aquarium containing artificial seawater without sulfide supplementation until March 2022. It was then placed in a Kautex bottle containing sediment with coffee filters and *Ulva rigida* (C.Agardh, 1823) powder for a week. The clam was sacrificed on 4 May 2022, and its gills were dissected and preserved in DNA/RNA shield (ZymoBiomics, USA).

##### DNA extraction and sequencing

DNA was extracted from half a gill using the TRIzol (Thermo Fisher Scientific) back-extraction protocol as described previously, optimized for high-molecular-weight DNA recovery [41]. Briefly, after phase separation with a back-extraction buffer, DNA was precipitated using isopropanol and glycogen, followed by an overnight incubation at −80°C. The resulting pellet was washed with sodium citrate in ethanol and 75% ethanol before resuspension in nuclease-free water. To ensure high purity, a final cleanup step was performed using the CleanAll Micro kit (Norgen Biotek, Canada) with DNA eluted in a pre-heated elution buffer. DNA was stored at 4°C until further processing. Input DNA was fragmented using g-TUBE (Covaris, USA) prior to library preparation. A PacBio ultra-low library was constructed and sequenced on the Sequel II system at the Max Planck Genome-Centre Cologne, generating high-fidelity data.

##### Quality filtering and genome assembly

To remove host and contamination reads, sequencing reads were mapped against a *Ca.* T. endolucinida Illumina MAG (P32_novoc.fa), which was assembled according to a previous study on lucinid symbionts [25], using seal.sh from BBMap v38.68 [49], and mapped reads were concatenated for downstream analyses. Concatenated reads were cleaned with reformat.sh from BBMap for a minimum length of 100 bp and a quality score of at least 33 (minlength = 100 qin = 33). Duplicate reads were removed using dedupe.sh from BBMap. Genome assembly was performed with Flye [69] using the parameters --asm-coverage 50 --genome-size 4.5 m. Quality control was conducted using CheckM2 v1.0.1 [59] to assess quality measures of the assembled genome. The genome was annotated using RAST [70] and EggNOG [71].

#### Carbon and nitrogen natural abundance analysis

Gill tissues of the lucinids *Cl. costata*, *Ct. imbricatula*, and *Co. orbicularis*, as well as the bivalve *Pinna carnea* (Gmelin, 1791), which is not known to host chemoautotrophic symbionts, of the family Pinnidae, all originating from the same seagrass meadow in Guadeloupe, were dried overnight at 45°C for isotope analyses. Dried gill tissues were decalcified in a desiccator containing 37% HCl overnight to remove all calcite-bound carbon and then homogenized into powder using a mortar and pestle. Approximately 1 mg of powdered gill tissues from each sample was weighed into tin boats (catalog number 200007074, Elementar Analysensysteme GmbH), and their carbon and nitrogen isotopic compositions (δ^13^C, δ^15^N) were measured using an elemental analyzer (Thermo Flash EA, 112 Series) coupled to a continuous-flow isotope ratio mass spectrometer (Delta Plus XP IRMS; Thermo Finnigan, Dreieich, Germany).

#### Statistical analysis of natural abundance isotopes

To analyze the effect of bivalve species on natural abundance isotope values (δ^13^C and δ^15^N), we first investigated if assumptions of normality and homogeneity of variance were met. For this, we used the Shapiro-Wilk test and Levene’s test. As assumptions were met for all groups, one-way ANOVA with *post hoc* TukeyHSD test was performed to test for significant differences in δ^13^C and δ^15^N separately among species. To visualize the results of the post-hoc comparisons, adjusted *P*-values from the Tukey HSD tests were extracted and displayed in generated boxplots of δ^13^C and δ^15^N by species. All analyses and visualizations were performed in R (version 4.4.0) using the packages car [72], dplyr [73], tidyverse [74], and ggplot2 [51].

### Localization of symbionts in clam gills using fluorescence *in situ* hybridization

We did fluorescence *in situ* hybridization (FISH) to visualize *Ca.* T. endolucinida in gill sections of *Co. orbicularis, Ct. imbricatula,* and *Cl. costata* (all Guadeloupe, Lesser Antilles, 2023). The specimens used to visualize the symbionts originated from the same sampling site but were collected at different time points (Table S1). A previously published species-specific probe targeting *Ca.* T. endolucinida with the fluorophore Cy3 conjugated to the 3′ end was used [24]. Dissected gills were preserved in 4% paraformaldehyde, dehydrated with an ethanol series (30%, 50%, 70%), and stored at 4°C until further use. The gills were embedded in paraffin wax, cut in 5 μm cross-sections, and subsequently mounted on SuperfrostPlus adhesion slides (Thermo Scientific) by the Histopathology Facility at Vienna BioCenter Core Facilities, Austria. The sections were dewaxed in Roti-Histol (Carl Roth) following the manufacturer’s instructions. Three microliters of probes were hybridized to the gill sections in a 35% formamide FISH buffer (20 μl; details on hybridization protocol are provided in Supplementary Methods). Following the hybridization, the samples were 4',6-diamidino-2-phenylindole (DAPI)-stained (1 μg/ml) and mounted in CitiFluor AF1 mounting media (EMS; details are provided in Supplementary Methods). Images were captured on a Leica TCS SP8 X confocal laser scanning microscope using 63× and 93× objectives (details are provided in Supplementary Methods).

### Metatranscriptome analysis

#### Raw read processing and transcript quantification

Adapters and PhiX contamination were removed from concatenated raw reads, and quality filtering was performed using the BBduk feature of BBmap v38.68 [49]. Ribosomal RNA reads were identified and sorted out using SortMeRNA v2.1b [75]. For this, two different databases were used: SILVA_138_SSU_tax_silva-db [76, 77] and a custom database containing lucinid-specific rRNA sequences (only_host_lucinid_rRNA_probes.fa) published previously [48]. To quantify transcript abundance, Kallisto v0.43.0 [78] was used with an indexed database from the PacBio reference genome of *Ca.* T. endolucinida (see the Nucleic Acid Extraction and Sequencing section). Transcripts per million (TPM) and estimated count values were extracted to generate a count matrix used for further analyses.

#### Differential gene expression analysis

Differential gene expression analysis was performed using the R package DESeq2 [79]. The dataset was prefiltered to remove genes with fewer than 10 counts across all samples. Pairwise comparisons were conducted between all host pairs (*Ct. imbricatula* vs. *Co. orbicularis*, *Ct. imbricatula* vs. *Cl. costata*, *Co. orbicularis* vs. *Cl. costata*) to identify differentially expressed genes using the following parameters: α = 0.05, the “greaterAbs” alternative hypothesis, and a log2 fold change threshold of 0.58, corresponding to a log2 1.5-fold change, to balance sensitivity and stringency. The independent hypothesis weighting method was employed for multiple testing correction [80]. Log fold change shrinkage was performed using the “ashr” method [81] to improve accuracy of fold change estimates. Significant differentially expressed genes were identified as those with an adjusted *P* value < 0.05. Heatmaps of differentially expressed genes were generated using variance-stabilized transformed read counts with the R package pheatmap v.1.0.12 [82]. Top 100 most highly expressed *Ca.* T. endolucinida genes in each host species were identified by calculating the median TPM values. Venny v.2.1 was used to visualize and identify the genes shared and uniquely significantly higher expressed specific to each host species [83]. Some differentially expressed genes with no specific annotation were further investigated using InterProScan [84].

## Results and discussion

### *Ca.* T. endolucinida associates with three different host species in a Caribbean seagrass meadow

**Figure 2:**
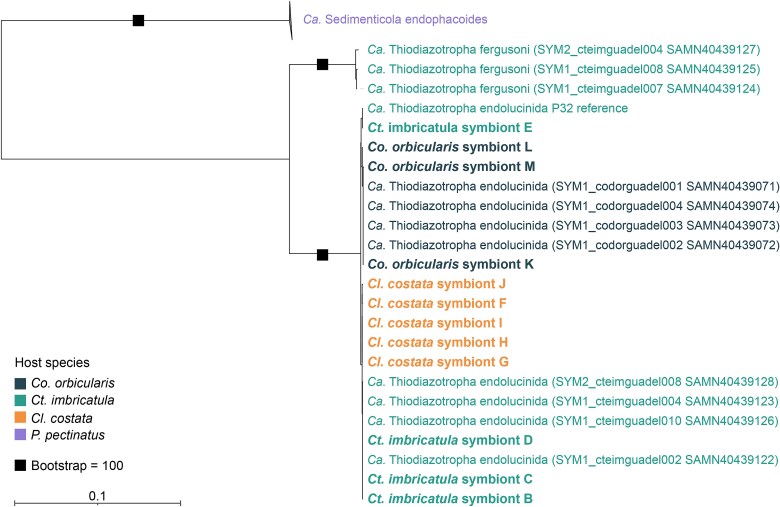
Relationship of new MAGs from this study to known lucinid symbiont species from Guadeloupe reveals that all samples belong to the symbiont species *Ca.* T. endolucinida. Maximum likelihood phylogenomic tree inferred from GTDB’s multiple sequence alignment using the best fit model Q.insect+F + G4. *Ca.* S. endophacoides was used as an outgroup. *Candidatus* T. endolucinida clade with MAGs sampled from Guadeloupe were expanded with newly placed MAGs from this study. Squares indicate bootstrap values of 100. Tree scale = 0.1.

The *T. testudinum* and *S. filiforme* seagrass meadow we sampled in Guadeloupe is mainly colonized by three different lucinid species: *Co. orbicularis, Ct. imbricatula*, and *Cl. costata,* which belong to two previously described subfamilies within the Lucinidae, Codakiinae, and Lucininae (Fig. 1F - G). Despite their phylogenetic divergence, these three lucinid species were previously reported to host the same symbiont species, *Ca.* T. endolucinida, although previous studies either did not include all three species or only focused on one of each species from this location [24, 25, 36]. To investigate host-symbiont pairings further at the genomic level, we sequenced metagenomes from three to five individuals of each species (*Ct. imbricatula n* = 4, *Cl. costata n* = 5, *Co. orbicularis n* = 3). From each of the metagenomes, a single high-quality metagenome-assembled genome (MAG) belonging to *Ca.* T. endolucinida could be assembled. These MAGs had >98% ANI and formed a monophyletic clade with previously sequenced *Ca.* T. endolucinida genomes [25] (Fig. 2). There may be fine-scale strain differences in the symbionts of each host species that were not visible at this level of analysis. Our analysis, however, could confirm that at the species level, the same symbiont species is hosted by all three lucinid species.

**Figure 3:**
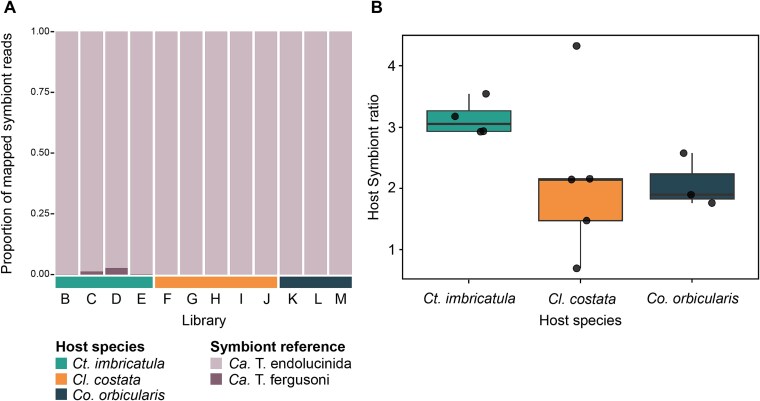
*Candidatus* T. endolucinida was dominant in all metagenomes and host:symbiont ratio maintained constant. (A) *Ca.* T. endolucinida dominated the symbiont community in all three lucinid host species. Relative abundance of *Ca.* T. endolucinida and *Ca. T. fergusoni* in *Ct. imbricatula* (samples B–E), *Cl. Costata* (samples F–J), and *Co. orbicularis* (samples K-M). (B) Host:symbiont ratio across all metagenomes per species *Ct. imbricatula* (*n* = 4), *Cl. costata* (*n* = 5), and *Co. orbicularis* (*n* = 3) shows no significant differences (pairwise TukeyHSD all adjusted *P* ≥ .31).

FISH with species-specific probes further confirmed the presence of this symbiont species in the gills of each species (Fig. 1C–E, S3). Symbiont abundance can differ within and between host species [24, 42]. The FISH images did not reveal any obvious and consistent differences in symbiont load between the three host species. To further investigate the symbiont load in the three species, we mapped the metagenomic reads to a reference MAG of *Ca.* T. endolucinida and to that of another symbiont species known to associate with lucinids in Guadeloupe, *Ca.* T. fergusoni [25]. The dominant symbiont species across all samples was *Ca.* T. endolucinida (Fig. 3A). Host-symbiont ratios across host species did not reveal significant differences (Fig. 3B; one-way ANOVA, *F*_2,9_ = 1.6, *P* = 0.26; pairwise Tukey HSD all adjusted *P* ≥  0.31).

### Gene expression by *Ca.* T. endolucinida varies depending on lucinid host species

We sequenced gill metatranscriptomes of three individuals of *Co. orbicularis* and five each of *Ct. imbricatula*, and *Cl. costata* sampled in the same seagrass bed on the same day in May 2023. As a reference used for transcriptomic analyses, we assembled a high-quality genome from long-read sequencing data from one *Ct. imbricatula* specimen. This MAG was 4.6 Mb long and assembled into four contigs, with 100% completeness and 0.1% contamination.

Analysis of the top 100 most highly transcribed *Ca.* T. endolucinida genes revealed a core set of 36 genes consistently highly expressed by symbionts associated with all three host species (Table 1). These genes were involved in key metabolic functions, including carbon and sulfur metabolism, stress response, RNA transcriptional regulation and translation, and oxidative stress protection. They highlight the essential physiological roles of the symbiont, which align with previous studies on lucinid symbionts [24, 25, 40, 42].

**Table 1:** Genes ranked among the top 100 highest transcribed and shared by the symbiont associated with all three hosts based on the median TPM values without hypothetical proteins.

Target ID	Description	Gene	*Co. orbicularis* associated (median TPM)	*Ct. imbricatula* associated (median TPM)	*Cl. costata* associated (median TPM)
fig|6666666.1078210.peg.1938	RNA-binding protein CsrA (carbon storage regulator)	*csrA*	85 248.5	78 449.9	83 987.0
fig|6666666.1078210.peg.3241	Sulfur transfer protein DsrC/TusE	*dsrC/tusE*	23 149.1	14 797.6	30 866.8
fig|6666666.1078210.peg.3960	Heat shock protein 10 kDa family chaperone GroES	*groES*	21 797.4	3324.43	30 700.2
fig|6666666.1078210.peg.2779	Cold shock protein of CSP family	*cspA/scpD*	11 051.1	38 508.6	20 704.4
fig|6666666.1078210.peg.846	Ribulose bisphosphate carboxylase small chain (EC 4.1.1.39)	*rbcS*	97 217.9	9600.54	19 133.9
fig|6666666.1078210.peg.580	Sulfur Carrier Protein	*tusA*	3899.09	8627.18	16 707.3
fig|6666666.1078210.peg.4209	Rubrerythrin		11 389.4	11 096.7	16 627.1
fig|6666666.1078210.peg.1615	RNA-binding protein	SLIRP/GR-RBP-like	14 064.8	17585.0	15 955.4
fig|6666666.1078210.peg.4049	Adenylylsulfate reductase beta-subunit (EC 1.8.99.2)	*aprB*	20 403.7	12 928.2	15 401.7
fig|6666666.1078210.peg.1640	Acyl carrier protein	*acp*	5755.12	14 695.5	11 226.5
fig|6666666.1078210.peg.3703	Cytochrome c-like domain superfamily	*cyt_c_ID*	9543.02	5008.51	8844.34
fig|6666666.1078210.peg.4029	Histone-like DNA-binding protein	Hist_DNA-bd	2906.01	2385.1	7614.07
fig|6666666.1078210.peg.3219	Sulfur oxidation cycle carrier protein SoxZ subunit	*soxZ*	4436.94	5043.88	7527.65
fig|6666666.1078210.peg.223	Gram-negative outer membrane porin	Gram-neg_bact_OMP	9815.19	7374.47	6956.14
fig|6666666.1078210.peg.3959	Heat shock protein 60 kDa family chaperone GroEL	*cpn60/groEL/TCP-1*	5530.88	1192.57	6531.75
fig|6666666.1078210.peg.4048	Adenylylsulfate reductase alpha-subunit (EC 1.8.99.2)	*aprA*	6611.64	4813.36	6470.93
fig|6666666.1078210.peg.2539	Hemerythrin HHE cation binding domain		19497.2	8622.24	6188.59
fig|6666666.1078210.peg.4093	Sulfur transfer protein DsrC/TusE	*dsrC/tusE*	8378.09	15535.3	5833.26
fig|6666666.1078210.peg.556	Histone-like DNA-binding protein	Hist_DNA-bd	3635.52	7213.49	5342.86
fig|6666666.1078210.peg.847	Ribulose bisphosphate carboxylase large chain (EC 4.1.1.39)	*rbcL*	25 842.7	2329.1	5005.98
fig|6666666.1078210.peg.3252	Sulfur transfer protein DsrC/TusE	*drsC/tusE*	6625.97	4994.64	4824.66
fig|6666666.1078210.peg.581	DsrE/DsrF/DrsH-like family		3034.73	2688.6	4507.25
fig|6666666.1078210.peg.3242	tRNA 5-methylaminomethyl-2-thiouridine synthase subunit TusB	*tusB*	2369.28	2164.78	4112.69
fig|6666666.1078210.peg.256	integral membrane protein	*DUF2282*	3251.47	5574.28	3735.63
fig|6666666.1078210.peg.197	Glutamate synthase [NADPH] large chain (EC 1.4.1.13)		2657.13	3810.47	3718.0
fig|6666666.1078210.peg.8	RNA-binding protein		5607.31	3348.94	3574.24
fig|6666666.1078210.peg.3218	Sulfur oxidation cycle carrier protein SoxY subunit	*soxY*	2770.76	1605.12	3232.06
fig|6666666.1078210.peg.706	NAD-dependent glyceraldehyde-3-phosphate dehydrogenase (EC 1.2.1.12)	4070.14	1331.93	2956.32	
fig|6666666.1078210.peg.2643	Cold shock protein of CSP family	*cspA*	2134.29	2584.39	2460.47
fig|6666666.1078210.peg.1429	RNA-binding protein Hfq	*hfq*	3940.57	5284.5	2299.0
fig|6666666.1078210.peg.3244	tRNA 5-methylaminomethyl-2-thiouridine synthase subunit TusD	*tusD*	1471.73	1499.51	2277.62
fig|6666666.1078210.peg.3988	Ribosome hibernation promoting factor Hpf	*hfp*	2519.5	4627.95	2088.13
fig|6666666.1078210.peg.1322	Protein of unknown function (DUF2909)		2277.62	4156.38	1840.8
fig|6666666.1078210.peg.423	SSU ribosomal protein S13p (S18e)	Ribosomal_uS13	2016.12	4227.31	1703.79
fig|6666666.1078210.peg.1916	Translation initiation factor 3		1257.09	1185.03	1577.57
fig|6666666.1078210.peg.2509	Type VI secretion system effector Hcp	T6SS_hcp	1162.94	1886.64	1538.12

**Figure 4:**
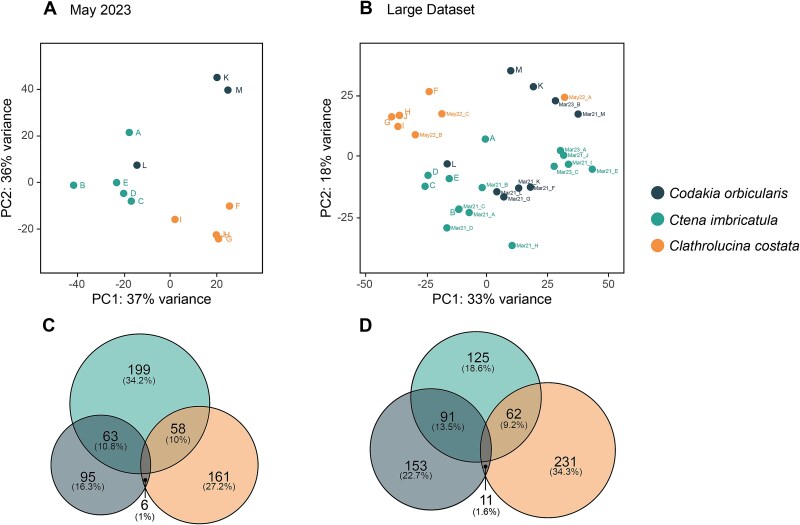
Symbiont transcriptomic patterns revealed distinct patterns related to host association. Principal component analysis (PCA) of variance-stabilized transformed count data for samples from May 2023 (A) and the large dataset (B). Clustering patterns indicate a strong influence of host species (*Co. orbicularis* (*n* = 3), *Ct. imbricatula* (*n* = 5), and *Cl. costata* (*n* = 5)) on transcriptional profiles. Venn diagrams for May 2023 (C) and the large dataset (D) illustrate the abundance and proportion of uniquely significantly more highly expressed and shared genes across host species.

Principal component analysis (PCA) of the variance-stabilized transcriptome count data revealed host species-level differentiation (Fig. 4A). Particularly, transcriptomes from *Ca.* T. endolucinida associated with *Cl. costata* were more distinctly separated from those associated with the other two hosts. In addition to the samples collected on the same day in May 2023, we also analyzed a larger dataset comprising samples collected from the same site but at different time points (details in Table S1). The PCA of the larger dataset (Fig. 4B) revealed more variability and greater overlap in symbiont transcriptional profiles when samples from different timepoints were included; however, some species-specific clustering was still visible. *Ctena imbricatula* samples show broader dispersion in comparison to *Cl. costata* and *Co. orbicularis*. Samples from symbionts associated with *Ct. imbricatula* and *Co. orbicularis* indicate a higher degree of overlap, potentially reflecting the more recent shared evolutionary history of these two host species (Fig. 1F and G) [21]. The samples did not cluster by sequencing batch, indicating that these patterns were not the result of technical artefacts but rather show biological differences in gene expression by *Ca.* T. endolucinida when in association with different host species, as observed differences are consistent over time.

Analysis of differential gene expression identified numerous symbiont genes that were more highly expressed when the symbionts were associated with a certain host (Fig. 4C, S2). For the May 2023 dataset, 95 genes were identified as upregulated in association with *Co. orbicularis*, 199 in *Ct. imbricatula*, and 161 in *Cl. costata*. Additionally, some genes were mutually expressed at higher levels by symbionts associated with two species than with the third: 63 in *Co. orbicularis* and *Ct. imbricatula* compared with *Cl. costata*, 58 in *Ct. imbricatula* and *Cl. costata* compared with *Co. orbicularis*, but only 6 in *Co. orbicularis* and *Cl. costata* compared with *Ct. imbricatula* (Fig. 4C). This is consistent with the PCA plot showing that at the global transcriptional level, symbionts of *Ct. imbricatula* clustered more closely with those from either *Co. orbicularis* and *Cl. costata*, whereas symbionts of *Co. orbicularis* and *Cl. costata* were more distinct from each other. In comparison to May 2023, more genes were differentially expressed by the symbiont in association with *Co. orbicularis* and *Cl. costata* but less in association with *Ct. imbricatula* in the larger dataset (Fig. 4D). The persistence of distinct transcriptomic profiles across hosts through time suggests that these factors are likely host species-specific rather than driven by environmental factors that change over time in shaping symbiont gene regulation, although further sampling would be required to determine how seasonal and geographical factors influence these patterns. Nevertheless, our dataset shows that there is overlap of symbiont transcriptomic profiles across host species; therefore, only genes that were exclusive and consistently differentially expressed across all time points were retained for downstream analyses (Table S3).

#### Differences in carbon fixation in *Co. orbicularis*-associated symbionts

**Figure 5:**
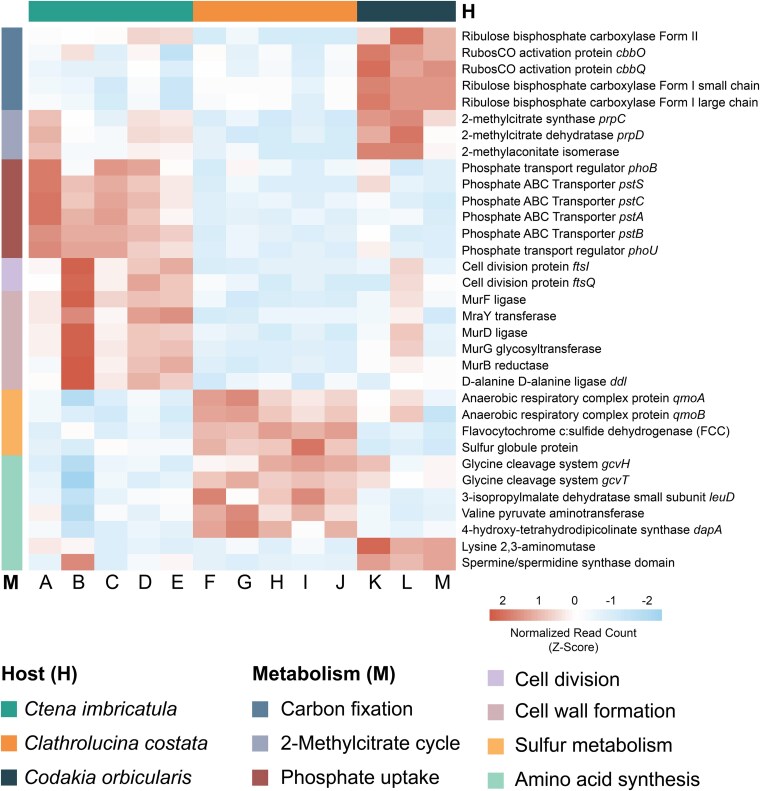
Differential expression analysis revealed that *Ca.* T. endolucinida adapts to distinct host environments. Exclusively upregulated genes from *Ca.* T. endolucinida associated with *Ct. imbricatula* (samples A–E), *Cl. costata* (samples F–J), and *Co. orbicularis* (samples K–M). Heatmap of *Z*-score-normalized read counts of selected genes, annotated with RAST and EggNOG, detected as differentially expressed by DESeq2.

In *Co. orbicularis*-associated *Ca.* T. endolucinida, genes involved in carbon fixation were expressed at significantly higher levels compared to *Ca.* T. endolucinida in both other host species (Fig. 5). Examples include genes encoding the small (*rbcS*) and large (*rbcL*) subunits of RuBisCO Form I, as well as the gene encoding RuBisCO Form II and the RuBisCO activation genes *cbbO* and *cbbQ*. Furthermore, transcripts for *rbcS*, *rbcL*, and *cbbQ* were among the top 40 most abundant based on median TPM values (Table S2), underscoring their critical role in the *Co. orbicularis* symbiosis. The presence in the genome and expression of both RubisCO Form I and Form II genes in *Ca.* T. endolucinida was previously reported by Osvatic *et al.* [24]. Our findings corroborate this, showing that both forms are transcribed, yet Form II RubisCO had much lower expression levels (median TPM 25.9) than Form I at the transcript level (median TPM of *rbcS* = 97 217.9 and *rbcL* = 25 842.7). Form I and Form II RubisCO have contrasting CO_2_ affinities, with Form I performing better at lower CO_2_ concentrations and Form II at higher CO_2_ concentrations [85, 86]. Form II is often found in bacteria-inhabiting environments with high concentrations of reduced compounds such as sulfide and with fluctuating redox conditions where electron acceptors such as O_2_ are limited [87]. The co-expression of both enzymes suggests that, although Form I may be the primary enzyme, the symbionts are adapted to fluctuating CO_2_ concentrations [88], which may enable the symbionts to rapidly adjust their carbon fixation efficiency in response to variable O_2_ and CO_2_ conditions. Another possibility is that O_2_ and CO_2_ gradients exist alongside the gill filament, from the apical to the basal end, causing distinct microenvironments with different symbiont expression profiles [89]. Spatial separation of metabolic subpopulations cannot be resolved in our metatranscriptomic analyses, and further experimental evidence is needed to investigate this. The higher expression of RubisCO activation genes *cbbO* and *cbbQ* in *Co. orbicularis*-associated symbionts further support increased symbiotic carbon fixation capabilities in this host species, as these genes play a crucial role in maintaining RubisCO in its active state [90].

**Figure 6:**
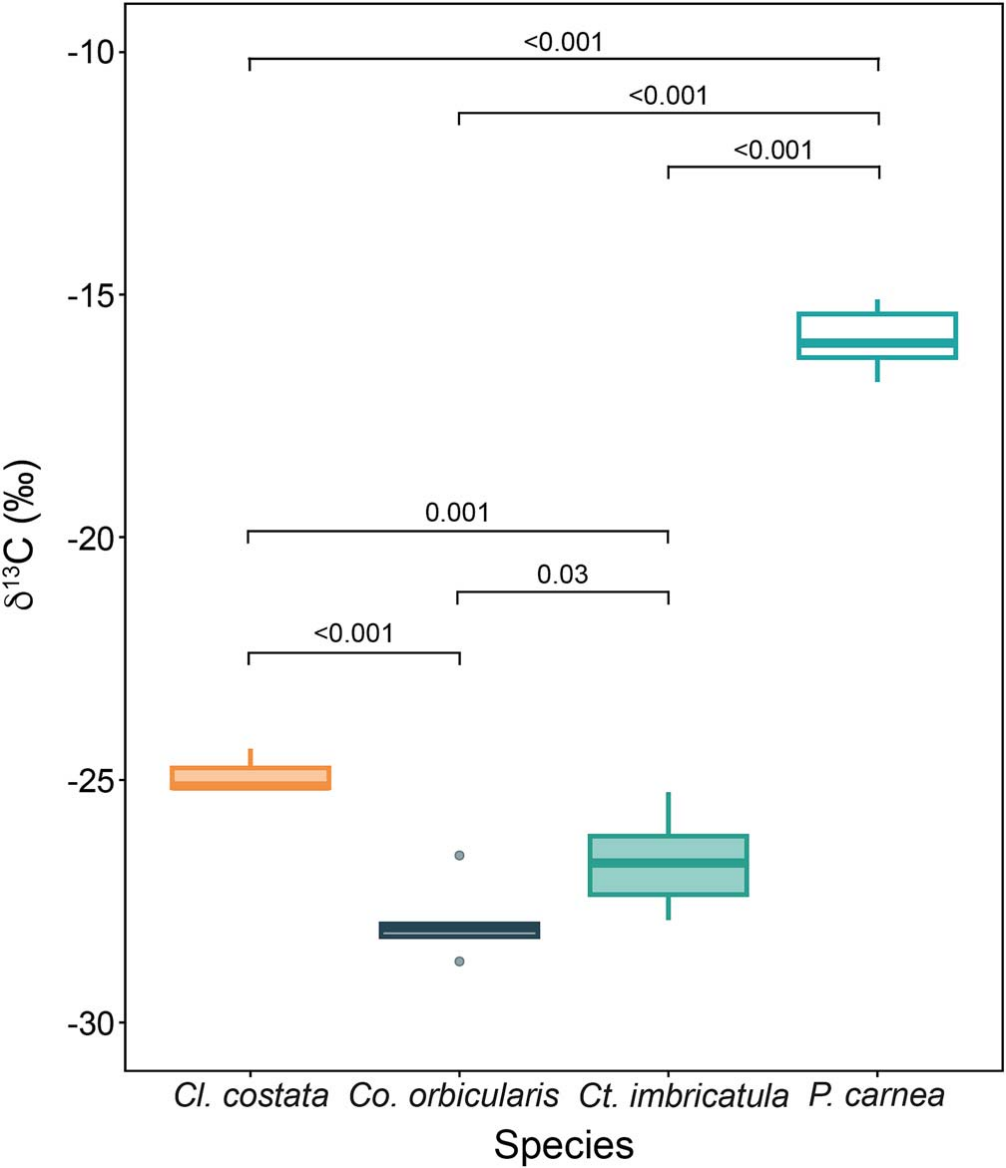
Natural abundance isotope data revealed gill tissues of *Co. orbicularis* were significantly most depleted in δ^13^C values. Carbon isotope ratios [‰] of gill tissue from lucinid species (full circles) *Co. orbicularis*, *Ct. Imbricatula*, and *Cl. costata*, and an asymbiotic control bivalve (empty circle) *P. carnea*. *P*-values are shown for each pair that was tested for significant differences in δ^13^C signatures. All samples were collected in the same seagrass bed in front of Îlet à Cochons, Guadeloupe.

To gain further insights into the dietary ecology of lucinid holobionts and to complement our transcriptome analyses, we measured carbon stable isotope compositions in the gills of the three lucinid species and the co-occurring pinnid bivalve *P. carnea,* which is not known to host chemosynthetic symbionts (here referred to as asymbiotic). The gill tissues of all three lucinid species were significantly more depleted in ^13^C compared to the asymbiotic *P. carnea* (mean difference = 9.0‰, *P* <  0.001 (*Cl. costata*), mean difference = 10.8‰, *P* <  0.001 (*Ct. imbricatula*), mean difference = 12.0‰, *P* <  0.001 (*Co. orbicularis*); Fig. 6, Fig. S1). The carbon isotope signature of the asymbiotic bivalve *P. carnea* corresponds to typical δ^13^C values described for filter-feeding organisms in seagrass environments [91]. In chemosynthetic symbioses, bacterial symbionts act as primary producers, fixing inorganic carbon and transferring organic carbon compounds to their hosts, the consumers [29, 92]. This depletion is the result of isotopic fractionation during chemosynthetic carbon fixation, where RubisCO discriminates against the heavier ^13^C isotope [93]. *Codakia orbicularis* gill tissue had the most negative δ^13^C values, which were significantly different from the other two hosts, *Ct. imbricatula* (mean difference = 1.2‰, *P* < 0.05) and *Cl. costata* (mean difference = 3.0‰, *P* <  0.001). This is consistent with the higher transcript abundance of RubisCO genes by its symbionts, further supporting the link between increased symbiotic carbon fixation and a more depleted gill isotopic signature. This pronounced depletion in ^13^C suggests that, in comparison to the other two lucinid hosts, *Co. orbicularis* relies more heavily on carbon derived from its symbionts’ CO_2_ fixation rather than on filter-feeding as a primary nutritional source [94]. This reliance highlights the possibility of diverse nutritional strategies within lucinids, where some host species may have evolved to depend more heavily on symbionts for carbon acquisition than others. Key knowledge on host morphology, physiology, and behavior that could affect filter-feeding activity, prey composition, or levels of mixotrophy needs further investigation. Future controlled experiments directly measuring carbon fixation and transfer rates, in addition to investigating filter-feeding activities of the hosts, could be used in the future to test this hypothesis.

#### Indications for higher symbiont turnover in *Ct. imbricatula–*associated symbionts

Several key genes involved in cell division (*ftsI, ftsQ*) and enzymes crucial for peptidoglycan biosynthesis (*murF*, *mraY*, *murD*, *murG*, *murB*, *ddl*) were significantly more highly expressed by *Ca.* T. endolucinida in *Ct. imbricatula*. These symbionts also had higher expression of genes encoding phosphate ABC transporters (*pstA, pstB, pstC, pstS*) and transport regulators (*phoB, phoU*). This pattern is consistent with the central role of phosphate in nucleic acid synthesis and phospholipid production, essential to bacterial growth and cell division [95, 96]. These gene expression patterns would be consistent with higher symbiont proliferation including DNA replication, cell growth, and division in *Ct. imbricatula* compared with the other two host species. Two different strategies have been described for nutrient transfer from symbionts to their bivalve hosts: a “milking” strategy where symbiont cells “leak” nutrients that are taken up by the hosts and “farming,” which involves intracellular digestion of symbiont cells [97–99]. In *Co. orbicularis* and *Ct. imbricatula* (previously identified as *Codakia orbiculata*), symbiont farming has been documented only during starvation periods [100, 101], where hosts progressively digest their endosymbionts to meet heightened energy demands, resulting in fewer symbiont cells in the gills, which could be beneficial, for example during breeding seasons. Based on the symbiont expression patterns in *Ct. imbricatula*, we hypothesize that this host may rely more on symbiont farming for nutrient transfer than the other two host species. All hosts investigated in this study breed during the same period (June-October, Gros personal observation); thus, our observations are inconsistent with symbiont digestion to provide energy for reproduction because this should be consistent across all three species. Additionally, enzymatic digestion by marine invertebrates, including lucinid species, hosting chemosynthetic symbionts has been shown regardless of reproduction or starvation [102, 103]. Although further experiments, for example proliferation assays, would be needed to confirm this hypothesis and explain the adaptive advantage of increased proliferation, if it is indeed happening specifically in *Ct. imbricatula*, then there may be major differences in how particular host species interact with their symbionts even within one host family.

#### Increased sulfide oxidation by symbionts of *Cl. costata*

Sulfide oxidation is a key metabolic process for lucinid symbionts [20, 41, 104], and accordingly, sulfur oxidation genes were prominent among the top 100 expressed genes in symbionts from all three species (Table 1). These include adenylylsulfate reductase (*aprAB*), encoding proteins that oxidize sulfite to adenosine-5′-phosphosulfate (APS), as well as genes for sulfur transfer proteins (*dsrC/tusE, dsrE/dsrF/dsrH, tusA*) and the sulfur carrier protein (*soxYZ*). However, symbionts associated with *Cl. costata* had higher expression levels of some pathways related to sulfide oxidation and energy conservation compared to those from the other hosts. These included genes encoding the anaerobic respiratory complex proteins (*qmoAB*), which facilitate electron transfer from adenylylsulfate reductase (*aprAB*) to the quinone pool in the electron transport chain (ETC) [105]. Furthermore, flavocytochrome c sulfide dehydrogenase (FCC), which catalyzes sulfide (H_2_S) oxidation to elemental sulfur (S^0^), transferring electrons to flavin groups and subsequently to cytochrome c, which then shuttles these electrons to the ETC, driving ATP production, was transcribed at higher levels [106]. In addition, a sulfur globule protein (*sgp*) gene was found to be more highly expressed in *Cl. costata* symbionts. This is a key protein for intracellular elemental sulfur storage in sulfide-oxidizing bacteria that ensures excess sulfur does not accumulate freely in the cells’ periplasm and provides a reserve for sulfur, which can be accessed for further oxidation when energy is needed [100, 107–109]. This indicates that *Ca.* T. endolucinida may have an increased need for sulfide storage, which also serves as an electron sink [110], when associated with *Cl. costata*. As sulfide oxidation by symbionts not only generates energy but also detoxifies excess sulfide in the host’s environment, symbiont sulfide oxidation will prevent sulfide toxicity that can inhibit cytochrome c oxidases and disrupt aerobic respiration by the host [111–113].

### Lack of host species-specific expression of known symbiont-host interaction mechanisms

Differential expression of host-symbiont interaction mechanisms such as toxins or secretion systems could be one way in which symbionts establish associations with distinct host species [114–117]. Intriguingly, we did not see any known host-microbe interaction mechanisms differentially expressed by symbionts in different host species. This could imply that identical mechanisms are employed by *Ca.* T. endolucinida to associate with all three host species. This is consistent with the observed flexibility of host-symbiont pairings in lucinid symbioses - there may be conserved host-microbe interaction mechanisms that all *Ca.* Thiodiazotropha utilize. Furthermore, one type VI secretion system component (effector Hcp) was among the top 100 shared expressed genes of *Ca.* T. endolucinida in association with all three hosts. This secretion system has been suggested to be involved in bacterial-eukaryotic cell interactions [118]. However, so little is known about the mechanisms underpinning host-microbe interactions in most symbioses, including lucinids, it is likely that currently unknown mechanisms encoded among the many hypothetical genes that were differentially expressed in each host could be involved in mediating molecular host-microbe interactions. Indeed, hypothetical genes made up to 27% of the differentially expressed genes (Table S3). These could be further investigated for potential roles in mediating species-specific host-microbe interactions.

### Fine-tuning transcriptional regulation in different host species may facilitate symbiont promiscuity and the ecological success of the lucinid symbioses

Lucinid clams thrive across a wide range of environments, from deep-sea hydrothermal vents to shallow coastal systems, showcasing the great adaptability of these holobionts [24, 25, 37, 40]. In such varying environments, we would expect different strategies to evolve for energy generation and nutritional exchange between host and symbiont. Indeed, in hydrothermal vent snails from the genus *Alviniconcha* specific associations between host and symbiont lineages correspond to differences in vent geochemistry [119, 120].

Our study, focusing on multiple co-occurring species in the same habitat, revealed an unexpected variability in potential host-driven strategies influencing the symbiont’s transcriptional profile. In the shared habitat of a seagrass bed, each host species seems to represent distinct microenvironments that drive host species-specific transcriptomic responses by a single symbiont species that can associate with all three.

Environmental measurements indicate that there could be a large imbalance between the availability of electron donors (high sulfide concentrations) and electron acceptors (low oxygen concentrations) in the sediments inhabited by lucinids [113, 121, 122]. If there is such an imbalance, we would expect them to have evolved strategies for dealing with excess electron donors. Our results would be consistent with each host differentially shaping its symbiont’s metabolism to deal with this excess, with for example symbionts in *Co. orbicularis* possibly directing reducing equivalents to CO_2_ through carbon fixation with RubisCO Form II and symbionts in *Cl. costata* possibly storing more elemental sulfur instead of oxidizing it further. As sulfide diffuses into the host tissues [23] and we did not see any sulfide-related genes differentially expressed in *Ct. imbricatula* symbionts, the host might be able to cope with high sulfide concentrations by, for example mitochondrial sulfide oxidation [123]. Availability of host genomes will enable comparison of host expression patterns in the future.

What could be driving the differences we observed in symbiont gene expression when in association with different host species? One possible explanation is that although they inhabit sediments in the same seagrass meadow, with their different sizes, they may each typically colonize different depths within the sediment, with *Co. orbicularis* borrowing deepest into the sediment due to its three to seven times larger size [45]. As sediment chemistry changes with depth, this could mean that the environment experienced by each clam species is the key factor. However, this is inconsistent with our experience of sampling these animals; we did not notice any species being typically found at a certain depth, although quantitative approaches would be needed to confirm these preliminary observations. Therefore, all hosts are presumed to experience similar environmental conditions, such as high sulfide levels, rapid oxygen depletion within the upper few millimeters of the sediment [122], and high levels of organic carbon [124]. An alternative explanation would be that the host, through its anatomy, physiology, and behavior, plays a much greater role than the external environment in shaping the microhabitats experienced by the symbionts. Although they seem anatomically very similar from the outside, differences between lucinid genera can be observed in regard to their gill filament structure with different types and abundances of various cells such as mucocytes, granule cells, and bacteriocytes [22]. Additionally, it has been shown that symbiont population regulation might be host species-specific [125]. The observed differences in gene expression patterns of the symbionts strongly suggest that the symbionts experience different internal conditions. These conditions could be due to physiological differences within the host body shaping the microenvironments of the symbionts, or environmental differences based on the different sizes and behaviors of the different host species. The difference in sizes could reflect the similarities observed in symbiont transcription patterns between *Ct. imbricatula* association and the other two host species, as *Ct. imbricatula* is typically mid-sized and could therefore provide more similar microenvironments in comparison to the other hosts, *Co. orbicularis* with *Cl. costata* [45]. Future studies are needed to investigate differences in host physiology in more depth.

Horizontal transmission of symbionts from the environment is known to enhance host adaptability and fitness as free-living symbiont populations can benefit from increased genetic diversity and frequent horizontal gene transfer events [5, 126]. Moreover, lucinids, such as *Ct. imbricatula,* have been shown to acquire symbionts from the environment throughout their entire life cycle [125, 127, 128]. Animals may gain significant advantages from associating with symbionts that exhibit a high degree of phenotypic flexibility. This symbiont plasticity is most likely the key feature allowing symbionts to promiscuously colonize and establish successful associations with diverse hosts. The broad range of metabolic functions encoded in the genomes of lucinid symbionts such as *Ca.* T. endolucinida has been hypothesized to play an important role in allowing these symbionts to survive within hosts, as well as in the environment as free-living stages. In this model, some functions would be upregulated in an animal host, and others in the environment. We show, however, that even among different host species from the same animal family, where conditions are likely more similar than compared to the outside environment, different symbiont pathways may be upregulated. Associations with distinct symbiont genotypes are one important factor underpinning the maintenance of plant and animal biodiversity. For example, in coral reef ecosystems, coral hosts thrive due to their association with diverse symbiotic dinoflagellate genotypes [129]. Our results show that even genetically identical symbionts could contribute to maintaining natural diversity through their phenotypic flexibility. Finally, the emerging availability of host genomes will allow such analyses in the future, which may reveal species-specific aspects of molecular host-microbe interactions such as differential expression of proteins for intracellular symbiont digestion or immune interactions and crosstalk [130].

## Supplementary Material

Supplementary_Tables_wrag065

Supplement_text_ckueck_et_al_wrag065

## Data Availability

Sequences have been deposited to the European Nucleotide Archive (ENA) under the Study PRJEB101735 with BioSample numbers SAMEA120468298-SAMEA120468329 (metagenomic and metatranscriptomic reads) and SAMEA120498306 - SAMEA120968719 and SAMEA120969524-SAMEA120969525 (MAGs). All scripts and description of bioinformatic and statistical analyses, as well as the code for plotting, can be found in the publicly available GitLab repository https://gitlab.mpi-bremen.de/ckueck/same_symbiont_different_host.

## References

[1] Henry LP, Bruijning M, Forsberg SKG et al. The microbiome extends host evolutionary potential. *Nat Commun* 2021;12:5141. 10.1038/s41467-021-25315-x 34446709 PMC8390463

[2] Dubilier N, Bergin C, Lott C. Symbiotic diversity in marine animals: the art of harnessing chemosynthesis. *Nat Rev Microbiol* 2008;6:725–40. 10.1038/nrmicro1992 18794911

[3] Voolstra CR, Ziegler M. Adapting with microbial help: microbiome flexibility facilitates rapid responses to environmental change. *BioEssays* 2020;42:e2000004. 10.1002/bies.202000004 32548850

[4] Marangon E, Laffy PW, Bourne DG et al. Microbiome-mediated mechanisms contributing to the environmental tolerance of reef invertebrate species. *Mar Biol* 2021;168:89. 10.1007/s00227-021-03893-0

[5] Wilkins LGE, Leray M, O’Dea A et al. Host-associated microbiomes drive structure and function of marine ecosystems. *PLoS Biol* 2019;17:e3000533. 10.1371/journal.pbio.3000533 31710600 PMC6874084

[6] Haine ER . Symbiont-mediated protection. *Proc Biol Sci* 2008;275:353–61. 10.1098/rspb.2007.1211 18055391 PMC2213712

[7] LaJeunesse. Diversity and community structure of symbiotic dinoflagellates from Caribbean coral reefs. *Mar Biol* 2002; 141:387–400. 10.1007/s00227-002-0829-2

[8] Chavez-Dozal AA, Gorman C, Lostroh CP et al. Gene-swapping mediates host specificity among symbiotic bacteria in a beneficial symbiosis. *PLoS One* 2014;9:e101691. 10.1371/journal.pone.0101691 25014649 PMC4094467

[9] Parker BJ, Hrček J, McLean AHC et al. Genotype specificity among hosts, pathogens, and beneficial microbes influences the strength of symbiont-mediated protection. *Evolution* 2017;71:1222–31. 10.1111/evo.13216 28252804 PMC5516205

[10] Bordenstein SR, Theis KR. Host biology in light of the microbiome: ten principles of holobionts and hologenomes. *PLoS Biol* 2015;13:e1002226. 10.1371/journal.pbio.1002226 26284777 PMC4540581

[11] Fisher RM, Henry LM, Cornwallis CK et al. The evolution of host–symbiont dependence. *Nat Commun* 2017;8:15973. 10.1038/ncomms15973 28675159 PMC5500886

[12] Doña J, Osuna-Mascaró C, Johnson KP et al. Persistence of single species of symbionts across multiple closely-related host species. *Sci Rep* 2019;9:17442. 10.1038/s41598-019-54015-2 31767919 PMC6877549

[13] Joseph MB, Stutz WE, Johnson PTJ. Multilevel models for the distribution of hosts and symbionts. *PLoS One* 2016;11:e0165768. 10.1371/journal.pone.0165768 27832124 PMC5104364

[14] Zimmermann J, Obeng N, Yang W et al. The functional repertoire contained within the native microbiota of the model nematode Caenorhabditis elegans. *ISME J* 2020;14:26–38. 10.1038/s41396-019-0504-y 31484996 PMC6908608

[15] Moran NA, Sloan DB. The hologenome concept: helpful or hollow? *PLoS Biol* 2015;13:e1002311. 10.1371/journal.pbio.1002311 26636661 PMC4670207

[16] Cavanaugh CM, Mckiness ZP, Newton I et al. Marine chemosynthetic symbioses. *The Prokaryotes* 2006;1:475–507. 10.1007/0-387-30741-9_18

[17] Sun J, Zhang Y, Xu T et al. Adaptation to deep-sea chemosynthetic environments as revealed by mussel genomes. *Nat Ecol Evol* 2017;1:0121. 10.1038/s41559-017-0121 28812709

[18] Eichinger I, Schmitz-Esser S, Schmid M et al. Symbiont-driven sulfur crystal formation in a thiotrophic symbiosis from deep-sea hydrocarbon seeps. *Environ Microbiol Rep* 2014;6:364–72. 10.1111/1758-2229.12149 24992535 PMC4232855

[19] Sun Y, Wang M, Zhong Z et al. Adaption to hydrogen sulfide-rich environments: strategies for active detoxification in deep-sea symbiotic mussels. *Gigantidas platifrons Sci Total Environ* 2022;804:150054. 10.1016/j.scitotenv.2021.150054 34509839

[20] Taylor JD, Glover EA. Lucinidae (Bivalvia)–the most diverse group of chemosymbiotic molluscs. *Zool J Linnean Soc* 2006;148:421–38. 10.1111/j.1096-3642.2006.00261.x

[21] Taylor JD, Glover EA, Yuen B et al. Closing the gap: a new phylogeny and classification of the chemosymbiotic bivalve family Lucinidae with molecular evidence for 73% of living genera. *J Molluscan Stud* 2022;88:eyac025. 10.1093/mollus/eyac025

[22] Taylor JD, Glover E. Biology, evolution and generic review of the chemosymbiotic bivalve family Lucinidae. *Zoological Journal of the Linnean Society* 2021;148:11–61.

[23] Dando P, Ridgway S, Spiro B. Sulphide ‘mining’ by lucinid bivalve molluscs: demonstrated by stable Sulphur isotope measurements and experimental models. *Mar Ecol Prog Ser* 1994;107:169–75. 10.3354/meps107169

[24] Osvatic JT, Wilkins LGE, Leibrecht L et al. Global biogeography of chemosynthetic symbionts reveals both localized and globally distributed symbiont groups. *Proc Natl Acad Sci USA* 2021;118:e2104378118. 10.1073/pnas.2104378118 34272286 PMC8307296

[25] Morel-Letelier I, Yuen B, Kück AC et al. Adaptations to nitrogen availability drive ecological divergence of chemosynthetic symbionts. *PLoS Genet* 2024;20:e1011295. 10.1371/journal.pgen.1011295 38820540 PMC11168628

[26] Distel DL, Felbeck H. Endosymbiosis in the lucinid clams *Lucinoma aequizonata, Lucinoma annulata* and *Lucina floridana*: a reexamination of the functional morphology of the gills as bacteria-bearing organs. *Mar Biol* 1987;96:79–86. 10.1007/BF00394840

[27] Durand P, Gros O. Bacterial host specificity of Lucinacea endosymbionts: interspecific variation in 16S rRNA sequences. *FEMS Microbiol Lett* 1996;140:193–8. 10.1111/j.1574-6968.1996.tb08335.x 8764482

[28] Durand P, Gros O, Frenkiel L et al. Phylogenetic characterization of sulfur-oxidizing bacterial endosymbionts in three tropical Lucinidae by 16S rDNA. *Mol Mar Biol Biotechnol* 1996;5:37–42.

[29] Herry A, Diouris M, Le Pennec M. Chemoautotrophic symbionts and translocation of fixed carbon from bacteria to host tissues in the littoral bivalve *Loripes lucinalis* (Lucinidae). *Mar Biol* 1989;101:305–12. 10.1007/BF00428126

[30] Felbeck H, Childress JJ, Somero GN. Calvin-Benson cycle and sulphide oxidation enzymes in animals from sulphide-rich habitats. *Nature* 1981;293:291–3. 10.1038/293291a0

[31] Duplessis MR, Dufour SC, Blankenship LE et al. Anatomical and experimental evidence for particulate feeding in *Lucinoma aequizonata* and *Parvilucina tenuisculpta* (Bivalvia: Lucinidae) from the Santa Barbara Basin. *Mar Biol* 2004;145:551–61. 10.1007/s00227-004-1350-6

[32] van der Geest M, Sall AA, Ely SO et al. Nutritional and reproductive strategies in a chemosymbiotic bivalve living in a tropical intertidal seagrass bed. *Mar Ecol Prog Ser* 2014;501:113–26. 10.3354/meps10702

[33] Gros O, Darrasse A, Durand P et al. Environmental transmission of a sulfur-oxidizing bacterial gill endosymbiont in the tropical lucinid bivalve *Codakia orbicularis*. *Appl Environ Microbiol* 1996;62:2324–30. 10.1128/aem.62.7.2324-2330.1996 8779569 PMC168012

[34] Gros O, Wulf-Durand P, Frenkiel L et al. Putative environmental transmission of sulfur-oxidizing bacterial symbionts in tropical lucinid bivalves inhabiting various environments. *FEMS Microbiol Lett* 1998;160:257–62. 10.1111/j.1574-6968.1998.tb12920.x

[35] Gros O, Duplessis MR, Felbeck H. Embryonic development and endosymbiont transmission mode in the symbiotic clam *Lucinoma aequizonata* (Bivalvia: Lucinidae). *Invertebr Reprod Dev* 1999;36:93–103. 10.1080/07924259.1999.9652683

[36] Giani NM, Lim SJ, Anderson LC et al. Variation in accessory and horizontal gene transfer-associated genes drives lucinid endosymbiont diversity. *FEMS Microbiol Ecol* 2025;101:fiaf074. 10.1093/femsec/fiaf074 40637797 PMC12278820

[37] Ratinskaia L, Malavin S, Zvi-Kedem T et al. Metabolically-versatile *Ca*. Thiodiazotropha symbionts of the deep-sea lucinid clam *Lucinoma kazani* have the genetic potential to fix nitrogen. *ISME Commun* 2024;4:ycae076. 10.1093/ismeco/ycae076 38873029 PMC11171427

[38] König S, Gros O, Heiden SE et al. Nitrogen fixation in a chemoautotrophic lucinid symbiosis. *Nat Microbiol* 2016;2:16193. 10.1038/nmicrobiol.2016.193 27775698

[39] Petersen JM, Kemper A, Gruber-Vodicka H et al. Chemosynthetic symbionts of marine invertebrate animals are capable of nitrogen fixation. *Nat Microbiol* 2016;2:16195. 10.1038/nmicrobiol.2016.195 27775707 PMC6872982

[40] Osvatic JT, Yuen B, Kunert M et al. Gene loss and symbiont switching during adaptation to the deep sea in a globally distributed symbiosis. *ISME J* 2023;17:453–66. 10.1038/s41396-022-01355-z 36639537 PMC9938160

[41] Sudo M, Osvatic J, Taylor JD et al. SoxY gene family expansion underpins adaptation to diverse hosts and environments in symbiotic sulfide oxidizers. *mSystems* 2024;9:e0113523. 10.1128/msystems.01135-23 38747602 PMC11237559

[42] Lim SJ, Alexander L, Engel AS et al. Extensive thioautotrophic gill endosymbiont diversity within a single *Ctena orbiculata* (Bivalvia: Lucinidae) population and implications for defining host–symbiont specificity and species recognition. *mSystems* 2019;4:10–1128. 10.1128/mSystems.00280-19 PMC6712303 31455638

[43] Amorim K, Loick-Wilde N, Yuen B et al. Chemoautotrophy, symbiosis and sedimented diatoms support high biomass of benthic molluscs in the Namibian shelf. *Sci Rep* 2022;12:9731. 10.1038/s41598-022-13571-w 35697901 PMC9192762

[44] Dubbs JM, Tabita FR. Regulators of nonsulfur purple phototrophic bacteria and the interactive control of CO_2_ assimilation, nitrogen fixation, hydrogen metabolism and energy generation. *FEMS Microbiol Rev* 2004;28:353–76. 10.1016/j.femsre.2004.01.002 15449608

[45] Taylor OD, Glover EA. Lucinid bivalves of Guadeloupe: diversity and systematics in the context of the tropical western Atlantic (Mollusca: Bivalvia: Lucinidae). *Zootaxa* 2016;4196:301–80. 10.11646/zootaxa.4196.3.1 27988661

[46] N Dawson, J Fischer, M Kuhn et al. qgis/QGIS: 3.40.11. 2025. Zenodo.

[47] Runfola D, Anderson A, Baier H et al. geoBoundaries: a global database of political administrative boundaries. *PLoS One* 2020;15:e0231866. 10.1371/journal.pone.0231866 32330167 PMC7182183

[48] Morel-Letelier I, Yuen B, Orellana LH et al. Seasonal transcriptomic shifts reveal metabolic flexibility of chemosynthetic symbionts in an upwelling region. *mSystems* 2025;10:e0168624. 10.1128/msystems.01686-24 40401909 PMC12172469

[49] Bushnell B . BBMap: A Fast, Accurate, Splice-Aware Aligner. Berkeley, CA (United States): Lawrence Berkeley National Lab. (LBNL), 2014, Report No.: LBNL-7065E.

[50] Gruber-Vodicka HR, Seah BKB, Pruesse E et al. PhyloFlash: rapid small-subunit rRNA profiling and targeted assembly from metagenomes. *mSystems* 2020;5:1–16. 10.1128/mSystems.00920-20 PMC7593591 33109753

[51] Wickham H Ggplot2: Elegant Graphics for Data Analysis. 2009. Springer, New York, NY, 10.1007/978-0-387-98141-3.

[52] Prjibelski A, Antipov D, Meleshko D et al. Using SPAdes DE novo assembler. *Curr Protoc Bioinformatics* 2020;70:1–29. 10.1002/cpbi.102 32559359

[53] Li H, Durbin R. Fast and accurate short read alignment with burrows-wheeler transform. *Bioinformatics* 2009;25:1754–60. 10.1093/bioinformatics/btp324 19451168 PMC2705234

[54] Danecek P, Bonfield JK, Liddle J et al. Twelve years of SAMtools and BCFtools. *Gigascience* 2021;10:giab008. 10.1093/gigascience/giab008 33590861 PMC7931819

[55] Kang DD, Li F, Kirton E et al. MetaBAT 2: an adaptive binning algorithm for robust and efficient genome reconstruction from metagenome assemblies. *PeerJ* 2019;7:e7359. 10.7717/peerj.7359 31388474 PMC6662567

[56] Graham ED, Heidelberg JF, Tully BJ. BinSanity: unsupervised clustering of environmental microbial assemblies using coverage and affinity propagation. *PeerJ* 2017;5:e3035. 10.7717/peerj.3035 28289564 PMC5345454

[57] Wu Y-W, Simmons BA, Singer SW. MaxBin 2.0: an automated binning algorithm to recover genomes from multiple metagenomic datasets. *Bioinformatics* 2016;32:605–7. 10.1093/bioinformatics/btv638 26515820

[58] Sieber CMK, Probst AJ, Sharrar A et al. Recovery of genomes from metagenomes via a dereplication, aggregation and scoring strategy. *Nat Microbiol* 2018;3:836–43. 10.1038/s41564-018-0171-1 29807988 PMC6786971

[59] Chklovski A, Parks DH, Woodcroft BJ et al. CheckM2: a rapid, scalable and accurate tool for assessing microbial genome quality using machine learning. *Nat Methods* 2023;20:1203–12. 10.1038/s41592-023-01940-w 37500759

[60] Jain C, Rodriguez-R LM, Phillippy AM et al. High throughput ANI analysis of 90K prokaryotic genomes reveals clear species boundaries. *Nat Commun* 2018;9:5114. 10.1038/s41467-018-07641-9 30504855 PMC6269478

[61] Chaumeil PA, Mussig AJ, Hugenholtz P et al. GTDB-Tk: a toolkit to classify genomes with the genome taxonomy database. *Bioinformatics* 2019;36:1925–7. 10.1093/bioinformatics/btz848 31730192 PMC7703759

[62] Chaumeil PA, Mussig AJ, Hugenholtz P et al. GTDB-Tk v2: memory friendly classification with the genome taxonomy database. *Bioinformatics* 38:5315–6. 36218463 10.1093/bioinformatics/btac672 PMC9710552

[63] Parks DH, Chuvochina M, Chaumeil PA et al. A complete domain-to-species taxonomy for bacteria and archaea. *Nat Biotechnol* 2020;38:1079–86. 10.1038/s41587-020-0501-8 32341564

[64] Parks DH, Chuvochina M, Waite DW et al. A standardized bacterial taxonomy based on genome phylogeny substantially revises the tree of life. *Nat Biotechnol* 2018;36:996–1004. 10.1038/nbt.4229 30148503

[65] Minh BQ, Schmidt HA, Chernomor O et al. IQ-TREE 2: new models and efficient methods for phylogenetic inference in the genomic era. *Mol Biol Evol* 2020;37:1530–4. 10.1093/molbev/msaa015 32011700 PMC7182206

[66] Hoang DT, Chernomor O, von Haeseler A et al. UFBoot2: improving the ultrafast bootstrap approximation. *Mol Biol Evol* 2018;35:518–22. 10.1093/molbev/msx281 29077904 PMC5850222

[67] Kalyaanamoorthy S, Minh BQ, Wong TKF et al. ModelFinder: fast model selection for accurate phylogenetic estimates. *Nat Methods* 2017;14:587–9. 10.1038/nmeth.4285 28481363 PMC5453245

[68] Letunic I, Bork P. Interactive tree of life (iTOL) v5: an online tool for phylogenetic tree display and annotation. *Nucleic Acids Res* 2021;49:W293–6. 10.1093/nar/gkab301 33885785 PMC8265157

[69] Kolmogorov M, Yuan J, Lin Y et al. Assembly of long, error-prone reads using repeat graphs. *Nat Biotechnol* 2019;37:540–6. 10.1038/s41587-019-0072-8 30936562

[70] Aziz RK, Bartels D, Best AA et al. The RAST server: rapid annotations using subsystems technology. *BMC Genomics* 2008;9:75. 10.1186/1471-2164-9-75 18261238 PMC2265698

[71] Huerta-Cepas J, Forslund K, Coelho LP et al. Fast genome-wide functional annotation through orthology assignment by eggNOG-mapper. *Mol Biol Evol* 2017;34:2115–22. 10.1093/molbev/msx148 28460117 PMC5850834

[72] Fox J, Weisberg S. An R Companion to Applied Regression, 3rd ed. 2019. Sage, Thousand Oaks CA. https://www.john-fox.ca/Companion/.

[73] Wickham H, François R, Henry L et al. Dplyr: a grammar of data manipulation. 2023.

[74] Wickham H, Averick M, Bryan J et al. Welcome to the tidyverse. *J Open Source Softw* 2019;4:1686. 10.21105/joss.01686

[75] Kopylova E, Noé L, Touzet H. SortMeRNA: fast and accurate filtering of ribosomal RNAs in metatranscriptomic data. *Bioinformatics* 2012;28:3211–7. 10.1093/bioinformatics/bts611 23071270

[76] Quast C, Pruesse E, Yilmaz P et al. The SILVA ribosomal RNA gene database project: improved data processing and web-based tools. *Nucleic Acids Res* 2013;41:D590–6. 10.1093/nar/gks1219 23193283 PMC3531112

[77] Yilmaz P, Parfrey LW, Yarza P et al. The SILVA and ‘all-species living tree project (LTP)’ taxonomic frameworks. *Nucleic Acids Res* 2014;42:D643–8. 10.1093/nar/gkt1209 24293649 PMC3965112

[78] Bray NL, Pimentel H, Melsted P et al. Near-optimal probabilistic RNA-seq quantification. *Nat Biotechnol* 2016;34:525–7. 10.1038/nbt.3519 27043002

[79] Love MI, Huber W, Anders S. Moderated estimation of fold change and dispersion for RNA-seq data with DESeq2. *Genome Biol* 15:550. 25516281 10.1186/s13059-014-0550-8 PMC4302049

[80] Ignatiadis N, Klaus B, Zaugg JB et al. Data-driven hypothesis weighting increases detection power in genome-scale multiple testing. *Nat Methods* 2016;13:577–80. 10.1038/nmeth.3885 27240256 PMC4930141

[81] Stephens M . False discovery rates: a new deal. *Biostatistics* 2017;18:275–94. 10.1093/biostatistics/kxw041 27756721 PMC5379932

[82] Kolde R . Pheatmap: pretty heatmaps. *Github* https://github.com/raivokolde/pheatmap.

[83] Collazos JCO . Venny 2.1.0. https://bioinfogp.cnb.csic.es/tools/venny/index.html. Accessed 14 Apr 2025.

[84] Jones P, Binns D, Chang H-Y et al. InterProScan 5: genome-scale protein function classification. *Bioinformatics* 2014;30:1236–40. 10.1093/bioinformatics/btu031 24451626 PMC3998142

[85] Robinson JJ, Stein JL, Cavanaugh CM. Cloning and sequencing of a form II ribulose-1,5-bisphosphate carboxylase/oxygenase from the bacterial symbiont of the hydrothermal vent tubeworm *Riftia pachyptila*. *J Bacteriol* 1998;180:1596–9. 10.1128/JB.180.6.1596-1599.1998 9515935 PMC107066

[86] Tabita FR . Microbial ribulose 1,5-bisphosphate carboxylase/oxygenase: a different perspective. *Photosynth Res* 1999;60:1–28. 10.1023/A:1006211417981

[87] Tabita FR, Satagopan S, Hanson TE et al. Distinct form I, II, III, and IV rubisco proteins from the three kingdoms of life provide clues about rubisco evolution and structure/function relationships. *J Exp Bot* 2008;59:1515–24. 10.1093/jxb/erm361 18281717

[88] Tabita FR, Hanson TE, Li H et al. Function, structure, and evolution of the RubisCO-like proteins and their RubisCO homologs. *Microbiol Mol Biol Rev* 2007;71:576–99. 10.1128/MMBR.00015-07 18063718 PMC2168653

[89] Petersen JM, Yuen B. The symbiotic ‘all-rounders’: partnerships between marine animals and chemosynthetic nitrogen-fixing bacteria. *Appl Environ Microbiol* 2021;87:e02129–0. 10.1128/AEM.02129-20 33355107 PMC8090883

[90] Tsai Y-CC, Lapina MC, Bhushan S et al. Identification and characterization of multiple rubisco activases in chemoautotrophic bacteria. *Nat Commun* 2015;6:8883. 10.1038/ncomms9883 26567524 PMC4660213

[91] Cui L, Jiang Z, Huang X et al. Identification of food sources in tropical seagrass bed food web using triple stable isotopes and fatty acid signatures. *Front Mar Sci* 2023;10:1–12. 10.3389/fmars.2023.1093181

[92] Distel DL, Felbeck H. Pathways of inorganic carbon fixation in the endosymbiont-bearing lucinid clam *Lucinoma aequizonata*. Part 1. Purification and characterization of the endosymbiotic bacteria. *J Exp Zool* 1988;247:1–10. 10.1002/jez.1402470102

[93] Ferrier-Pagès C, Leal MC. Stable isotopes as tracers of trophic interactions in marine mutualistic symbioses. *Ecol Evol* 2019;9:723–40. 10.1002/ece3.4712 30680151 PMC6342181

[94] Duperron S, Gaudron SM, Rodrigues CF et al. An overview of chemosynthetic symbioses in bivalves from the North Atlantic and Mediterranean Sea. *Biogeosciences* 2013;10:3241–67. 10.5194/bg-10-3241-2013

[95] Romano S, Schulz-Vogt HN, González JM et al. Phosphate limitation induces drastic physiological changes, virulence-related gene expression, and secondary metabolite production in *Pseudovibrio* sp. strain FO-BEG1. *Appl Environ Microbiol* 2015;81:3518–28. 10.1128/AEM.04167-14 25769826 PMC4407226

[96] Billini M, Hoffmann T, Kühn J et al. The cytoplasmic phosphate level has a central regulatory role in the phosphate starvation response of *Caulobacter crescentus*. *Commun Biol* 2024;7:772. 10.1038/s42003-024-06469-y 38926609 PMC11208175

[97] Yuen B, Polzin J, Petersen JM. Organ transcriptomes of the lucinid clam *Loripes orbiculatus* (Poli, 1791) provide insights into their specialised roles in the biology of a chemosymbiotic bivalve. *BMC Genomics* 2019;20:820. 10.1186/s12864-019-6177-0 31699041 PMC6836662

[98] Kádár E, Davis SA, Lobo-da-Cunha A. Cytoenzymatic investigation of intracellular digestion in the symbiont-bearing hydrothermal bivalve *Bathymodiolus azoricus*. *Mar Biol* 2008;153:995–1004. 10.1007/s00227-007-0872-0

[99] Zheng P, Wang M, Li C et al. Insights into deep-sea adaptations and host–symbiont interactions: a comparative transcriptome study on *Bathymodiolus* mussels and their coastal relatives. *Mol Ecol* 2017;26:5133–48. 10.1111/mec.14160 28437568

[100] Caro A, Got P, Bouvy M et al. Effects of long-term starvation on a host bivalve (*Codakia orbicularis*, Lucinidae) and its symbiont population. *Appl Environ Microbiol* 2009;75:3304–13. 10.1128/AEM.02659-08 19346359 PMC2681646

[101] König S, Le Guyader H, Gros O. Thioautotrophic bacterial endosymbionts are degraded by enzymatic digestion during starvation: case study of two lucinids *Codakia orbicularis* and *C. orbiculata*. *Microsc Res Tech* 2015;78:173–9. 10.1002/jemt.22458 25429862

[102] Boetius A, Felbeck H. Digestive enzymes in marine invertebrates from hydrothermal vents and other reducing environments. *Mar Biol* 1995;122:105–13. 10.1007/BF00349283

[103] Liberge M, Gros O, Frenkiel L. Lysosomes and sulfide-oxidizing bodies in the bacteriocytes of *Lucina pectinata*, a cytochemical and microanalysis approach. *Mar Biol* 2001;139:401–9. 10.1007/s002270000526

[104] Alcaraz CM, Séneca J, Kunert M et al. Sulfur-oxidizing symbionts colonize the digestive tract of their lucinid hosts. *ISME J* 2024;18:wrae200. 10.1093/ismejo/wrae200 39388223 PMC11549920

[105] Ramos AR, Keller KL, Wall JD et al. The membrane QmoABC complex interacts directly with the dissimilatory adenosine 5′-phosphosulfate reductase in sulfate reducing bacteria. *Front Microbiol* 2012;3:137. 10.3389/fmicb.2012.00137 22536198 PMC3333476

[106] Tikhonova TV, Lilina AV, Osipov EM et al. Catalytic properties of flavocytochrome c Sulfide dehydrogenase from haloalkaliphilic bacterium *Thioalkalivibrio paradoxus*. *Biochemistry (Mosc)* 2021;86:361–9. 10.1134/S0006297921030111 33838635

[107] Prange A, Engelhardt H, Trüper HG et al. The role of the sulfur globule proteins of Allochromatium vinosum: mutagenesis of the sulfur globule protein genes and expression studies by real-time RT-PCR. *Arch Microbiol* 2004;182:165–74. 10.1007/s00203-004-0683-3 15340792

[108] Brune DC . Isolation and characterization of sulfur globule proteins from *Chromatium vinosum* and *Thiocapsa roseopersicina*. *Arch Microbiol* 1995;163:391–9. 10.1007/BF00272127 7575095

[109] Lechaire JP, Frébourg G, Gaill F et al. In situ characterization of Sulphur in gill-endosymbionts of the shallow water lucinid *Codakia orbicularis* (Linné, 1758) by high-pressure cryofixation and EFTEM microanalysis. *Mar Biol* 2008;154:693–700. 10.1007/s00227-008-0962-7

[110] Duplessis MR, Ziebis W, Gros O et al. Respiration strategies utilized by the gill endosymbiont from the host lucinid *Codakia orbicularis* (Bivalvia: Lucinidae). *Appl Environ Microbiol* 2004;70:4144–50. 10.1128/AEM.70.7.4144-4150.2004 15240294 PMC444781

[111] Chou P-H, Hu MY, Guh Y-J et al. Cellular mechanisms underlying extraordinary sulfide tolerance in a crustacean holobiont from hydrothermal vents. *Proc Biol Sci* 2023;290:20221973. 36629118 10.1098/rspb.2022.1973 PMC9832567

[112] Thiermann F, Vismann B, Giere O. Sulphide tolerance of the marine nematode *Oncholaimus campylocercoidesa* result of internal Sulphur formation? *Mar Ecol Prog Ser* 2000;193:251–9. 10.3354/meps193251

[113] van der Heide T, Govers LL, de Fouw J et al. A three-stage symbiosis forms the foundation of seagrass ecosystems. *Science* 2012;336:1432–4. 10.1126/science.1219973 22700927

[114] Costa TRD, Felisberto-Rodrigues C, Meir A et al. Secretion systems in Gram-negative bacteria: structural and mechanistic insights. *Nat Rev Microbiol* 2015;13:343–59. 10.1038/nrmicro3456 25978706

[115] Bontemps Z, Paranjape K, Guy L. Host-bacteria interactions: ecological and evolutionary insights from ancient, professional endosymbionts. *FEMS Microbiol Rev* 2024;48:fuae021. 10.1093/femsre/fuae021 39081075 PMC11338181

[116] Sayavedra L, Kleiner M, Ponnudurai R et al. Abundant toxin-related genes in the genomes of beneficial symbionts from deep-sea hydrothermal vent mussels. *elife* 2015;4:e07966. 10.7554/eLife.07966 26371554 PMC4612132

[117] Sato Y, Jang S, Takeshita K et al. Insecticide resistance by a host-symbiont reciprocal detoxification. *Nat Commun* 2021;12:6432. 10.1038/s41467-021-26649-2 34741016 PMC8571283

[118] Peng Y, Wang X, Shou J et al. Roles of Hcp family proteins in the pathogenesis of the porcine extraintestinal pathogenic *Escherichia coli* type VI secretion system. *Sci Rep* 2016;6:26816. 10.1038/srep26816 27229766 PMC4882540

[119] Beinart RA, Sanders JG, Faure B et al. Evidence for the role of endosymbionts in regional-scale habitat partitioning by hydrothermal vent symbioses. *Proc Natl Acad Sci USA* 2012;109:E3241–50. 10.1073/pnas.1202690109 23091033 PMC3511114

[120] Beinart RA, Luo C, Konstantinidis KT et al. The bacterial symbionts of closely related hydrothermal vent snails with distinct geochemical habitats show broad similarity in chemoautotrophic gene content. *Front Microbiol* 2019;10:1818. 10.3389/fmicb.2019.01818 31474946 PMC6702916

[121] Reynolds LK, Berg P, Zieman JC. Lucinid clam influence on the biogeochemistry of the seagrass *Thalassia testudinum* sediments. *Estuar Coasts* 2007;30:482–90. 10.1007/BF02819394

[122] Borum J, Pedersen O, Greve TM et al. The potential role of plant oxygen and sulphide dynamics in die-off events of the tropical seagrass, *Thalassia testudinum*. *J Ecol* 2005;93:148–58. 10.1111/j.1365-2745.2004.00943.x

[123] Hildebrandt TM, Grieshaber MK. Redox regulation of mitochondrial sulfide oxidation in the lugworm, Arenicola marina. *J Exp Biol* 2008;211:2617–23. 10.1242/jeb.019729 18689415

[124] Sánchez-Rojas MA, Ruiz-Fernández AC, van Tussenbroek BI et al. Quantifying organic carbon burial rates and stocks in seagrass meadow sediments influenced by sargassum-brown tides. *Mar Environ Res* 2025;204:106875. 10.1016/j.marenvres.2024.106875 39631320

[125] Elisabeth NH, Caro A, Césaire T et al. Comparative modifications in bacterial gill-endosymbiotic populations of the two bivalves *Codakia orbiculata* and *Lucina pensylvanica* during bacterial loss and reacquisition. *FEMS Microbiol Ecol* 2014;89:646–58. 10.1111/1574-6941.12366 24939560

[126] López-Madrigal S, Gil R. Et tu, brute? Not even intracellular mutualistic symbionts escape horizontal gene transfer. *Genes (Basel)* 2017;8:247. 10.3390/genes8100247 28961177 PMC5664097

[127] Gros O, Elisabeth NH, Gustave S et al. Plasticity of symbiont acquisition throughout the life cycle of the shallow-water tropical lucinid *Codakia orbiculata* (Mollusca: Bivalvia): symbiont acquisition in lucinid clams. *Environ Microbiol* 2012;14:1584–95. 10.1111/j.1462-2920.2012.02748.x 22672589

[128] Elisabeth NH, Gustave SDD, Gros O. Cell proliferation and apoptosis in gill filaments of the lucinid *Codakia orbiculata* (Montagu, 1808) (Mollusca: Bivalvia) during bacterial decolonization and recolonization. *Microsc Res Tech* 2012;75:1136–46. 10.1002/jemt.22041 22438018

[129] Ng MS, Soon N, Afiq-Rosli L et al. Highly diverse Symbiodiniaceae types hosted by corals in a global hotspot of marine biodiversity. *Microb Ecol* 2024;87:92. 10.1007/s00248-024-02407-x 38987492 PMC11236936

[130] McKenna V, Archibald JM, Beinart R et al. The aquatic Symbiosis genomics project: probing the evolution of symbiosis across the tree of life. *Wellcome Open Res* 2021;6:254. 10.12688/wellcomeopenres.17222.1 40438199 PMC12117321

