# Supplementary material for: Host species-specific gene expression by a widespread and flexible chemosynthetic symbiont
Source: ISME J. 2026 Mar 24;20(1):wrag065. doi: 10.1093/ismejo/wrag065 (PMC13137333; doi:10.1093/ismejo/wrag065)
Supplement: Supplement_text_ckueck_et_al_wrag065 [file supplement_text_ckueck_et_al_wrag065.docx]

## Supplementary Information

## Host species-specific gene expression by a widespread and flexible chemosynthetic symbiont

**A. Carlotta Kück, Lukas Leibrecht, Isidora Morel-Letelier, Olivier Gros, Laetitia G. E. Wilkins, Benedict Yuen-Simović and Jillian M. Petersen**

## Supplementary Methods

### Fluorescence *in situ* hybridisation

#### Fixation

Gills were dissected from *Codakia orbicularis, Ctena imbricatula* and *Clathrolucina costata* (all Guadeloupe, French Antilles, 2023) and fixed in a 4% paraformaldehyde, 10% sucrose, pH 7.4, 0.01 M PBS solution overnight at 4°C. The gills were then washed three times with a 10% sucrose, pH 7.4, 0.01 M PBS solution for 10 min during each wash. Washed gills were then dehydrated in 30%, 50%, and 70% ethanol for 10 min each and stored in 70% ethanol at 4°C until embedding. Gill pieces were embedded in 1% low-melting agarose prior to dehydration and paraffin embedding.

#### Sectioning and dewaxing

The paraffin-embedded gills of the clams were cross-sectioned at 5 µm by the Histopathology Facility at Vienna BioCenter Core Facilities, Austria and subsequently mounted on SuperfrostPlus adhesion slides (Thermo Scientific, USA). Sections were then dried in a horizontal position at room temperature overnight. Sections were dewaxed by three consecutive 10 min washes with Roti-Histol (Carl Roth), followed by two 10 min washes with 99% Ethanol and three 5 min washes of 1X PBS. Dewaxed sections were dried using compressed air.

#### FISH

For the hybridization chamber half a Kimwipe was folded and placed into a 50mL Falcon tube and soaked with 2mL 35% FISH buffer (see below). After circling gill sections with a PAP-pen (Kisker Biotech GmbH & Co. KG, Germany), 15-20µL of FISH hybridization buffer were applied to the sections. Subsequently, 3µL (15µmol) of the probes were added and the slides were put into the hybridization chamber and incubated for 3h at 46°C in dark conditions. Subsequently, the slides were washed by incubating at 48°C in the corresponding washing buffer. To ensure removal of any remaining Formamide the slides were subsequently dipped in MilliQ water and dried with compressed air.

#### DAPI staining

20µL of DAPI (1µg/mL in PBS) were applied to the sections and incubated for 15 minutes at room temperature in a dark box. Remaining DAPI solution was removed by dipping the slides three times in pre-cooled MilliQ water. After slides were completely dried an antifade mounting medium CitiFluor AF1 (EMS) was added to the slides.

#### Microscopy

Images were captured with a Leica TCS SP8 X confocal laser scanning microscope using a 63X objective (HC PL APO CS2 63x/1.30 GLYC) for *Codakia orbicularis* and a 93X objective (HC PL APO CS2 93x/1.30 GLYC) for *Ctena imbricatula* and *Clathrolucina costata*. The Leica software LASX was used for image acquisition and post-procession if necessary.

#### FISH hybridization buffer 35%, 1mL (µL if not differently stated):

| 5M NaCl | 180 |
| --- | --- |
| 1M Tris/HCl (pH=7.0) | 20 |
| MilliQ | 449 |
| Formamide (4%, deionized) | 350 |
| 10% SDS | 1 |

#### Washing buffer 35%, 50mL (mL if not differently stated):

| 5M NaCl | 0.70 |
| --- | --- |
| 1M Tris/HCl (pH=7.0) | 1 |
| 0.5M EDTA (ph=8.0) | 0.5 |
| 10% SDS | 50 µL |
| MilliQ | 47.8 |

## Supplementary Results and Discussion

### Differences in carbon utilization in *Co. orbicularis* associated symbionts

Genes encoding key enzymes involved in the 2-methylcitrate cycle [1, 2], including 2-Methylcitrate Synthase (*prpC*), 2-Methylcitrate Dehydratase (*prpD*), and 2-Methylaconitate Isomerase, were significantly more highly expressed by *Ca*. T. endolucinida in *Co. orbicularis*. This pathway converts propionyl-CoA into pyruvate and succinate, which can be used for energy production [3]. This pathway offers potential benefits for the symbionts, such as propionate detoxification (reviewed in [4]), or additional energy production, as it supplements the glyoxylate pathway and the TCA cycle for ATP generation with pyruvate and succinate [5, 6]. Additionally, it allows bacteria to use propionate and fatty acids as additional carbon sources, enhancing metabolic flexibility [1, 7]. The increased expression of genes involved in the 2-methylcitrate cycle in *Co. orbicularis* symbionts possibly suggests greater carbon demands in this holobiont, possibly due to differences in the host’s size, physiology or growth rate compared to the other two lucinid hosts. Furthermore, this expression pattern could reflect differences in availability of key compounds for the 2-methylcitrate cycle due to differences of substrate provision by the three hosts investigated. The ability to use alternative carbon sources like propionate and fatty acids may provide an advantage in seagrass sediments, where organic carbon is abundant and diverse but nutrient availability can be highly variable [8–10]. Although all three host species inhabit the same nutrient-variable environment, *Co. orbicularis* may have a higher need for carbon, necessitating a broader metabolic repertoire in its symbiont, as a strategy to buffer against periods of low inorganic carbon availability or competition with other organisms. Overall, the increased expression of genes enabling the utilization of multiple carbon sources via the methylcitrate cycle alongside efficient CO_2_ fixation makes *Ca.* Thiodiazotropha endolucinida associated with *Co. orbicularis* a highly versatile primary producer, possibly enhancing its host’s survival.

#### Increased amino acid metabolism in symbionts of *Cl. costata*

Symbionts associated with *Cl. costata* exhibited the broadest range of highly expressed genes related to amino acid transport and metabolism (Fig S2). Symbionts associated with *Ct. imbricatula* showed no increased expression of genes related to amino acid metabolism, while *Co. orbicularis* symbionts showed higher expression of genes involved in lysine transformation (lysine 2,3-aminomutase) and polyamine biosynthesis (spermine/spermidine synthase domain). In association with *Cl. costata,* symbionts showed increased expression of genes encoding components of the glycine cleavage system (*gcvH, gcvT*) and genes related to the biosynthesis of leucine (*leuD*), valine (valine pyruvate aminotransferase) and lysine (4-hydroxy-tetrahydrodipicolinate synthase *dapA*). Bivalves, including lucinids, are incapable of synthesizing essential amino acids, such as leucine, lysine, valine and others, relying instead on external sources to meet their nutritional needs (26, 27). The glycine cleavage system (GCS) catalyses the reversible degradation of glycine into carbon dioxide, a methylene group and ammonia. This process serves dual roles in bacterial metabolism: in glycine breakdown, it provides ammonia as a nitrogen source for biosynthetic pathways; in the reverse direction, the GCS synthesizes glycine by recycling nitrogen (28, 29). Symbionts of *Cl. costata*, the smallest lucinid among the three hosts investigated, may encounter environmental conditions that increase dependence on amino acid biosynthesis or nitrogen recycling, potentially due to reduced host dietary intake or elevated metabolic demands.

## References

1. Claes WA, Pühler A, Kalinowski J. Identification of two *prpDBC* gene clusters in *Corynebacterium glutamicum* and their involvement in propionate degradation via the 2-methylcitrate cycle. *J Bacteriol* 2002; **184**: 2728–2739.

2. Lim SJ, Davis B, Gill D, Swetenburg J, Anderson LC, Engel AS, et al. Gill microbiome structure and function in the chemosymbiotic coastal lucinid *Stewartia floridana*. *FEMS Microbiol Ecol* 2021; **97:** fiab042.

3. Limenitakis J, Oppenheim RD, Creek DJ, Foth BJ, Barrett MP, Soldati-Favre D. The 2-methylcitrate cycle is implicated in the detoxification of propionate in *Toxoplasma gondii*. *Mol Microbiol* 2013; **87**: 894–908.

4. Dolan SK, Wijaya A, Geddis SM, Spring DR, Silva-Rocha R, Welch M. Loving the poison: the methylcitrate cycle and bacterial pathogenesis. *Microbiology* 2018; **164**: 251–259.

5. Huang Z, Wang Q, Khan IA, Li Y, Wang J, Wang J, et al. The methylcitrate cycle and its crosstalk with the glyoxylate cycle and tricarboxylic acid cycle in pathogenic fungi. *Molecules* 2023; **28**: 6667.

6. Horswill AR, Escalante-Semerena JC. *Salmonella typhimurium* LT2 catabolizes propionate via the 2-methylcitric acid cycle. *J Bacteriol* 1999; **181**: 5615–5623.

7. Upton AM, McKinney JD. Role of the methylcitrate cycle in propionate metabolism and detoxification in Mycobacterium smegmatis. *Microbiology* 2007; **153**: 3973–3982.

8. Brenner CL, Valdez SR, Zhang YS, Shaver EC, Hughes BB, Silliman BR, et al. Sediment carbon storage differs in native and non-native Caribbean seagrass beds. *Mar Environ Res* 2024; **194**: 106307.

9. Nava-Félix TF, van Tussenbroek BI, Mateo-Cid LE, Ruiz-Fernández AC. Blue carbon stocks in seagrass tissues: A case study from the northern Mexican Caribbean. *Estuar Coast Shelf Sci* 2025; **323**: 109395.

10. Samper-Villarreal J, Moya-Ramírez J, Cortés J. Megaherbivore exclusion led to more complex seagrass canopies and increased biomass and sediment Corg pools in a tropical meadow. *Front Mar Sci* 2022; **9**: 945783.

## Supplementary Figures


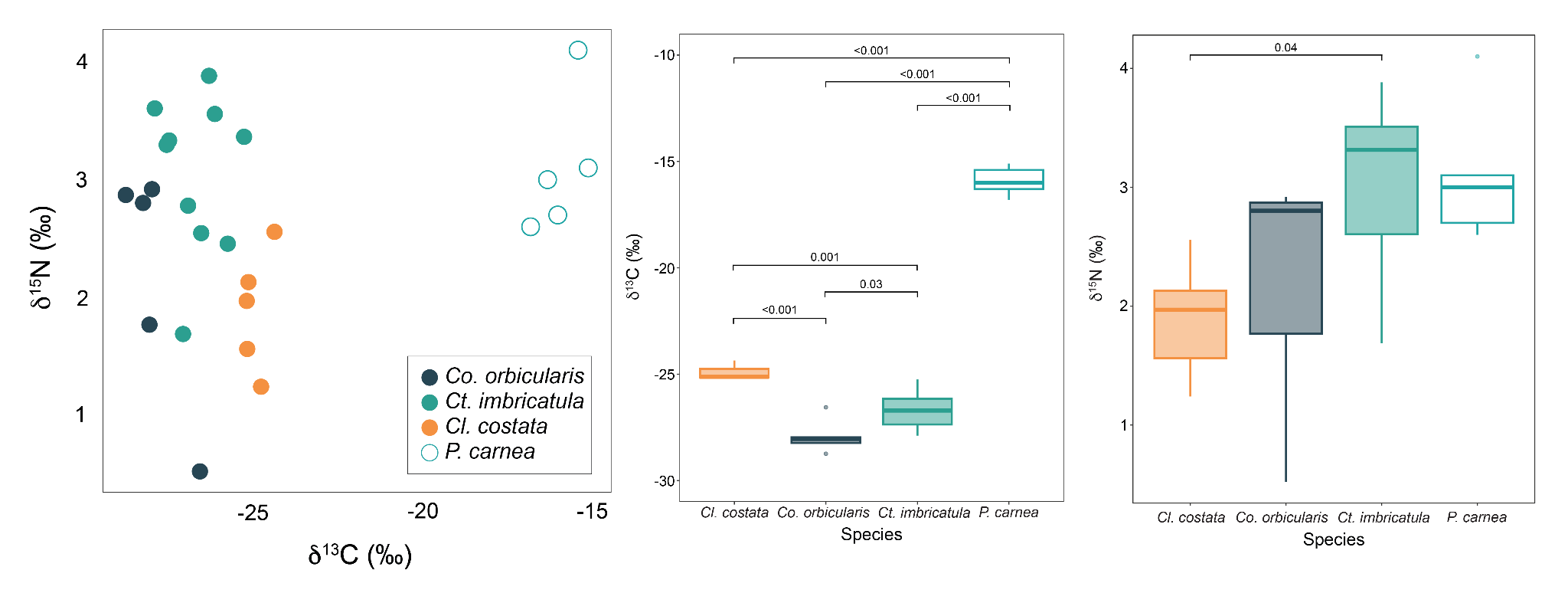


**Fig S1.** Natural abundance isotopes and separate boxplots of carbon and nitrogen isotope ratios [‰] with added *P* values where significant differences between isotope values were detected. Gill tissues from lucinid species (full circles) *Co. orbicularis* (dark blue), *Ct. imbricatula* (turquoise), and *Cl. costata* (yellow), and an asymbiotic control bivalve (empty circle) *P. carnea* (light blue). *P* values are shown for each pair that was tested for significant differences in δ13C signatures. All samples were collected in the same seagrass bed in front of Îlet à Cochons, Guadeloupe.


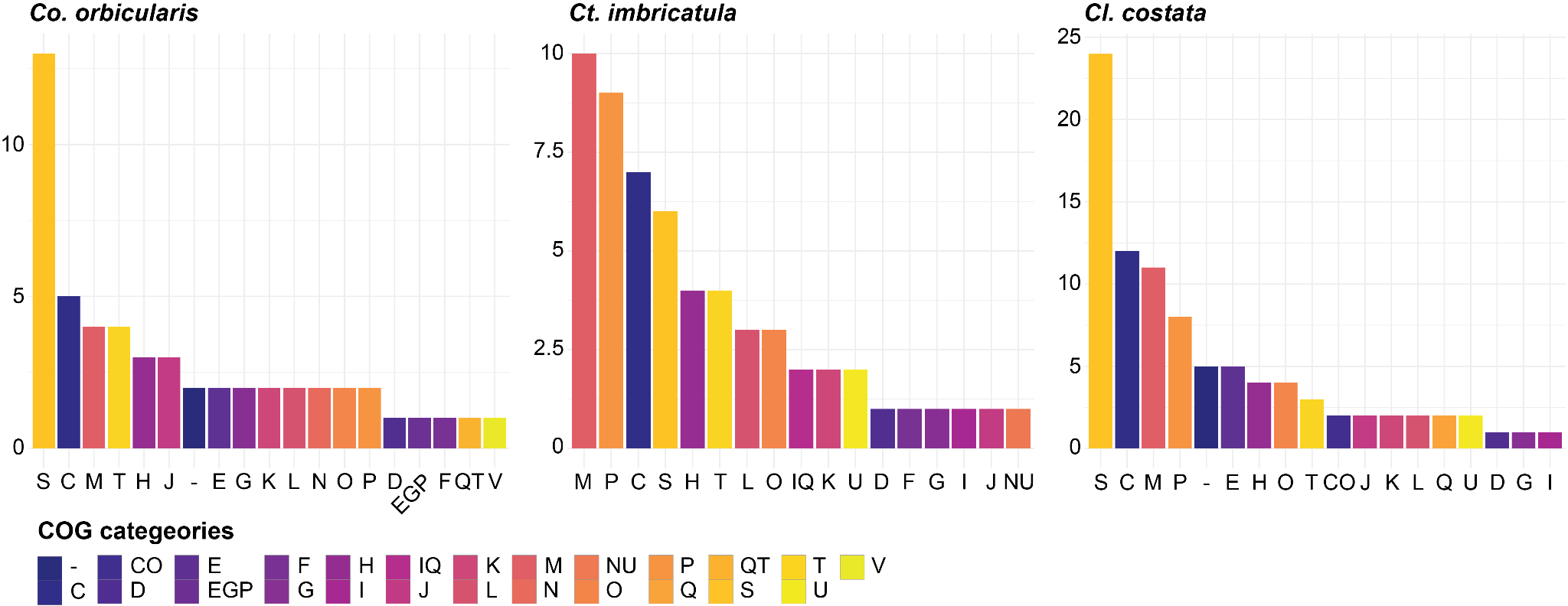


**Fig S2.** COG categories of differentially expressed genes of *Ca.* Thiodiazotropha endolucinida in association with the three different hosts.


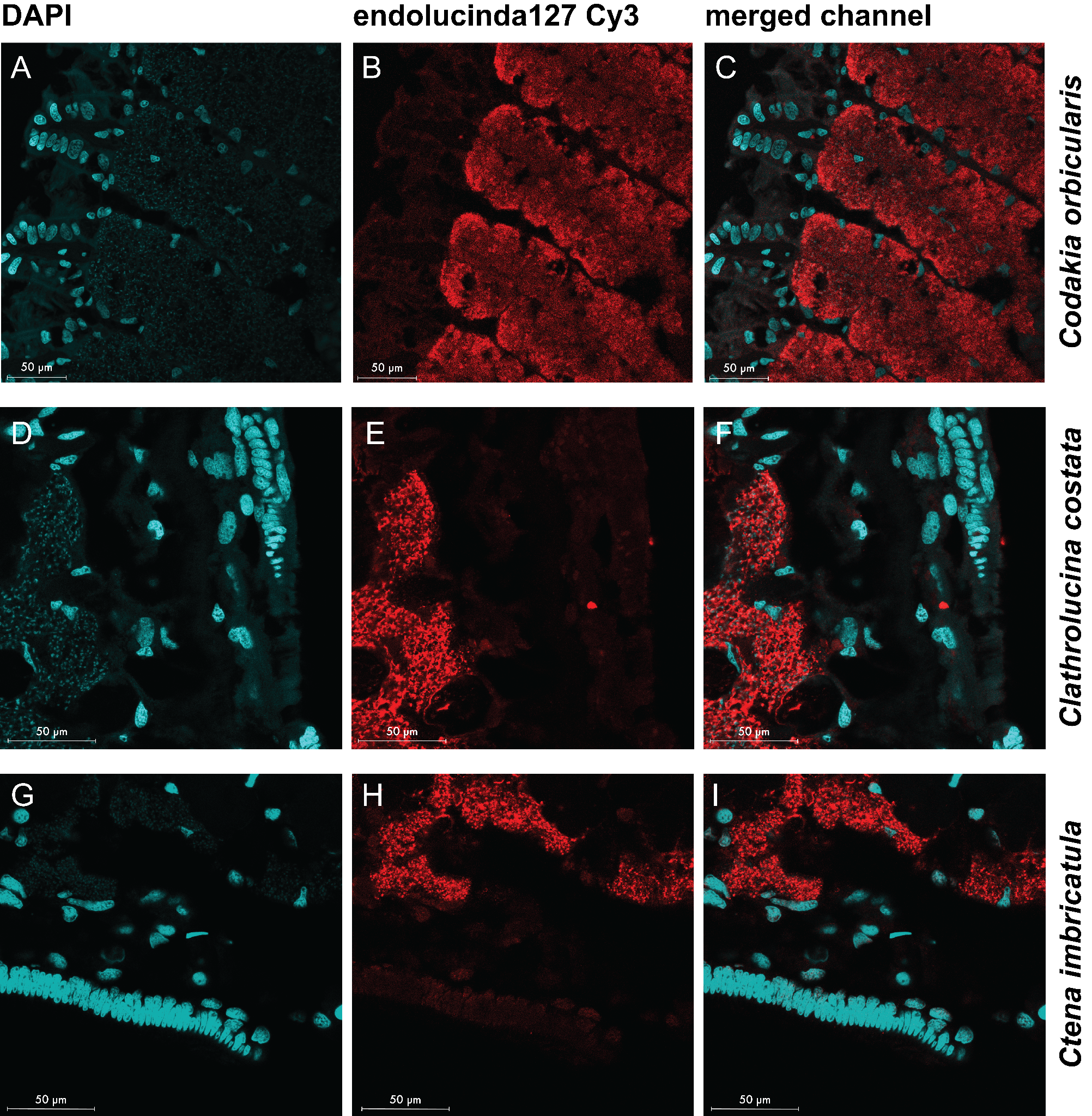


**Fig. S3.** Spatial distribution of Ca. T. endolucinida within gill filaments of *Co. orbicularis* (A-C, *Cl. costata* (D-F), and *Ct. imbricatula* (G-I) separated by DAPI (A,D,G) the symbiont species-specific probe endolucinida127 (B,E,H) and the overlay (C,F,I).
